# Photodegradation and photostabilization of polymers, especially polystyrene: review

**DOI:** 10.1186/2193-1801-2-398

**Published:** 2013-08-23

**Authors:** Emad Yousif, Raghad Haddad

**Affiliations:** Department of Chemistry, College of Science, Al-Nahrain University, Baghdad, Iraq

## Abstract

Exposure to ultraviolet (UV) radiation may cause the significant degradation of many materials. UV radiation causes photooxidative degradation which results in breaking of the polymer chains, produces free radical and reduces the molecular weight, causing deterioration of mechanical properties and leading to useless materials, after an unpredictable time. Polystyrene (PS), one of the most important material in the modern plastic industry, has been used all over the world, due to its excellent physical properties and low-cost. When polystyrene is subjected to UV irradiation in the presence of air, it undergoes a rapid yellowing and a gradual embrittlement. The mechanism of PS photolysis in the solid state (film) depends on the mobility of free radicals in the polymer matrix and their bimolecular recombination. Free hydrogen radicals diffuse very easily through the polymer matrix and combine in pairs or abstract hydrogen atoms from polymer molecule. Phenyl radical has limited mobility. They may abstract hydrogen from the near surrounding or combine with a polymer radical or with hydrogen radicals. Almost all synthetic polymers require stabilization against adverse environmental effects. It is necessary to find a means to reduce or prevent damage induced by environmental components such as heat, light or oxygen. The photostabilization of polymers may be achieved in many ways. The following stabilizing systems have been developed, which depend on the action of stabilizer: (1) light screeners, (2) UV absorbers, (3) excited-state quenchers, (4) peroxide decomposers, and (5) free radical scavengers; of these, it is generally believed that excited-state quenchers, peroxide decomposers, and free radical scavengers are the most effective. Research into degradation and ageing of polymers is extremely intensive and new materials are being synthesized with a pre-programmed lifetime. New stabilizers are becoming commercially available although their modes of action are sometimes not thoroughly elucidated. They target the many possible ways of polymer degradation: thermolysis, thermooxidation, photolysis, photooxidation, radiolysis etc. With the goal to increase lifetime of a particular polymeric material, two aspects of degradation are of particular importance: Storage conditions, and Addition of appropriate stabilizers. A profound knowledge of degradation mechanisms is needed to achieve the goal.

**Electronic supplementary material:**

The online version of this article (doi:10.1186/2193-1801-2-398) contains supplementary material, which is available to authorized users.

## Introduction

Exposure to ultraviolet (UV) radiation may cause significant degradation of many materials. UV radiation causes photooxidative degradation which results in breaking of the polymer chains, produces radicals and reduces the molecular weight, causing deterioration of mechanical properties and leading to useless materials, after an unpredictable time.

Polystyrene (PS), one of the most important materials from the modern plastic industry, has been used all over the world, due to its excellent physical properties and low-cost. Rabie et al. (2008).

When polystyrene is subjected to UV irradiation in the presence of air, it undergoes a rapid yellowing and a gradual embrittlement Geuskens and David (1975).

The mechanism of PS photolysis in the solid state (film) depends on the mobility of radicals in the polymer matrix and their bimolecular recombination. Hydrogen radicals diffuse very easily through the polymer matrix and combine in pairs or abstract hydrogen atoms from polymer molecule. The phenyl radical has limited mobility. It may abstract hydrogen from the near surrounding or combine with a polymer radical or with hydrogen radicals.

Almost all synthetic polymers require stabilization against adverse environmental effects. It is necessary to find a means to reduce or prevent damage induced by environmental components such as heat, light or oxygen Rabek and Ranby ([Bibr CR90][Bibr CR91]).

The photostabilization of polymers may be achieved in many ways. The following stabilizing systems have been developed, which depend on the action of stabilizer: (1) light screeners, (2) UV absorbers, (3) excited-state quenchers, (4) peroxide decomposers, and (5) radical scavengers; of these, it is generally believed that excited-state quenchers, peroxide decomposers, and radical scavengers are the most effective Yousif et al. (2013).

Research into degradation and ageing of polymers is extremely intensive and new materials are being synthesized with a pre-programmed lifetime. New stabilizers are becoming commercially available although their modes of action are sometimes not been thoroughly elucidated. They target the many possible ways of polymer degradation: thermolysis, thermooxidation, photolysis, photooxidation, radiolysis, etc. With the goal for increasing lifetime of a particular polymeric material, two aspects of degradation are of particular importance: storage conditions, and addition of appropriate stabilizers. A profound knowledge of degradation mechanisms is needed to achieve the goal.

### Definitions & historical aspects of photodegradation of polymers

Polymers are a broad class of materials which are made from repeating units of smaller molecule called monomers. Polymers can be natural in origin such as the lignin of tree branches. Other polymers are termed synthetic, because they are made by humans from naturally-occurring materials. Example such as polyester and polystyrene. Polymers are useful because of their strength and durability in many applications.

One of the disadvantages of using polymers is that they degrade when they are used in high temperature conditions or in outdoor applications. When polymers are used in outdoor applications, the environment negatively influences the servicelife. This process is called weathering (Zweifel 1998; Wypych 2008).

The term *degradation* of macromolecules denotes all processes which lead to a decline of polymer properties. It may eventually involve physical processes, such as polymer recrystallization, or denaturation of protein structures. Chemical processes related to degradation may lead to a reduction of average molar mass due to macromolecular chain bond scission or to an increase of molar mass due to crosslinking rendering the polymer insoluble.

The term *ageing* of polymers is usually associated with long-term changes of polymer properties under the conditions of weathering and may involve any of the above processes Strlic & Kolar (2005).

A wide variety of synthetic and naturally occurring high polymers absorb solar ultraviolet radiation and undergo photolytic, photooxidative, and thermooxidative reactions that result in the degradation of the material (Ayako and Hirose 1999; Scott 2000; Valkoa et al. 2001).

In recent years, the use of polymeric materials has rapidly increased but it is well established that rapid photodegradation of these materials is possible when they are exposed to natural weathering (Guillet 1985; Hamid 2000; Rabek 1996; Bottino et al. 2003). This is a serious issue, with economic and environmental implications and therefore a large effort is focused on understanding the changes that occur at molecular level and the degradation kinetics. Following different routes, UV radiation causes a photooxidative degradation which results in breaking of the polymer chains, produces radical and reduces the molecular weight, causing deterioration of mechanical properties and leading to useless materials, after an unpredictable time (Bottino et al. 2003; Gardella 1988).

**Figure 1:**
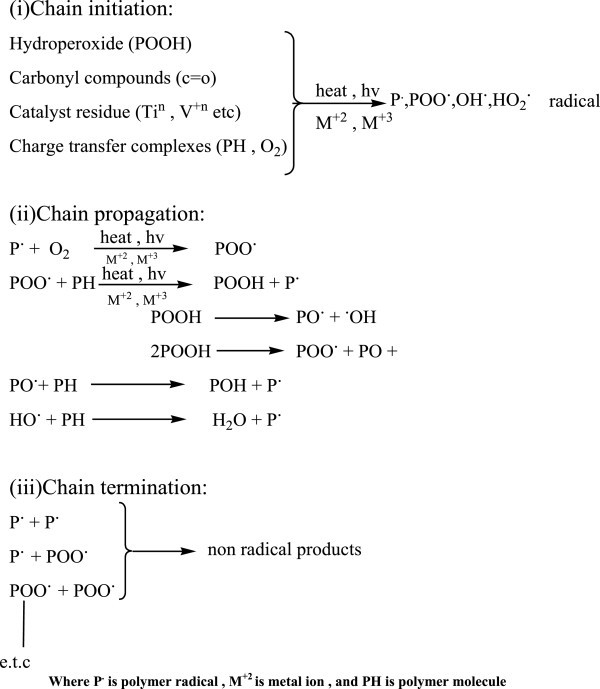
**General oxidation & photooxidation in polymers, (Rabek** 1994 **).**

Exposure to ultraviolet, UV, radiation may cause the significant degradation of many materials. Damage by UV radiation is commonly the main reason for the discoloration of dyes and pigments, weathering, yellowing of plastics, loss of gloss and mechanical properties (cracking), sun burnt skin, skin cancer, and other problems associated with UV light. The manufacturers of paints, plastics, contact lenses, and cosmetics have a great interest in offering products that remain unaltered for long periods under conditions of light exposure (Galdi *et al.*^2010^; Pospisil et al. 2006; Bojinov & Grabchev 2005; Goldshtein and Margel 2011), (Figure 1).

Most of the common polymers used in such applications contain photostabilizers to reduce photodamage and to ensure acceptable life times under outdoor exposure conditions.

The use of plastics in building applications is popular in the developing world because of the low cost and the ease of use of plastic components compared to the conventional metal, glass, mortar, wood and other materials. Plastics are used in other products such as outdoor furniture, fishing gear, and marine craft, which are also routinely used outdoors (Andrady 1997; Sampers 2002).

Solar radiation reaching the surface of the earth is characterized by wave lengths from approximately 295 up to 2500 nm. The solar radiation classified as UV-B (280 – 315 nm) has an energy of 426 – 380 KJ mol^-1^. Fortunately, the higher energetic part of UV-B; 280 – 295 nm, is filtered by the stratosphere and does not reach the earth’s surface, UV-A (315 – 400 nm), has energy between 389 and 300 KJ mol^-1^ and is less harmful for organic materials than UV-B. Visible (400 – 760 nm). and infrared (760 – 2500nm.) Pospisil and Nespurek (2000).

Photooxidation of organic materials is a major cause of irreversible deterioration for a large number of substances. It is responsible not only for the loss of physical properties of plastics, rubber, but also for foodstuffs Grassie and Scott (1985). In most polymers photooxidative degradation may be induced by UV radiation or catalytic process (or both) and can be accelerated at elevated temperature.

### Factors causing the photodegradation

Generally, many factors are responsible for causing photodegradation of polymeric materials. They may be divided into two categories: Schnabel (1981)

I) Internal impurities, which may contain chromophoric groups that are introduced into macromolecules during polymerization processing and storage; they include:
  a- Hydroperoxide.
  b- Carbonyl.
  c- Unsaturated bonds (C=C).
  d- Catalyst residue.
  e- Charge–transfer (CT) complexes with oxygen.
II) External impurities, which may contain chromophoric groups, are:
  a- Traces of solvents, catalyst, …. etc.
  b- Compounds from a polluted urban atmosphere and smog, e.g. polynuclear hydrocarbons such as naphthalene and anthracene in polypropylene and polybutadiene.
  c- Additives (pigments, dyes, thermal stabilizers, photostabilizers, …. etc.).
  d- Traces of metals and metal oxides from processing equipment and containers, such as Fe, Ni or Cr.

### Types of polymers degradation

The degradation of polymers usually starts at the outer surface and penetrates gradually into the bulk of the material Blaga (1980).

Polymer degradation can be caused by heat (thermal degradation), light (photodegradation), ionizing radiation (radio degradation), mechanical action, or by fungi, bacteria, yeasts, algae, and their enzymes (biodegradation). The deleterious effects of weathering on polymers generally has been ascribed to a complex set of processes in which the combined action of UV. light and oxygen predominant. The overall light-initiated process in the presence of oxygen generally is referred to as oxidative photodegradation or photooxidation. A pure thermal effect in possible because oxygen is always present and so the process is thermaloxidative degradation Feldman (2002).

There are many different modes of polymer degradation. These are very similar since they all involve chemical reactions that result in bond scission. These modes are: Schnabel (1981).

### Chemical degradation

Chemical degradation refers exclusively to processes, which are induced under the influence of chemical reagent (e.g. acids, bases, solvents reactive gases, etc.) Weidner et al. (1996).

### Thermal degradation

Thermal degradation refers to the case where the polymer, at elevated temperatures, starts to undergo chemical changes without the simultaneous involvement of another compound Guaita et al. (1985).

### Biodegradation

Biologically initiated degradation also is strongly related to chemical degradation as far as microbial attack is concerned. Microorganisms produce variety of enzymes which are capable of reaction with natural and synthetic polymers Dindar and Icli, (2001).

### Radiolytic degradation

When polymeric materials are subjected to high energy radiation (e.g. gamma radiation) changes are observed on their molecular structure, mainly chain scission, which leads to reduction in molar mass Vinhas et al. (2003).

### Mechanical degradation

This generally, refers to macroscopic effects brought about under the influence of shear forces. These forces result in the formation of macro radicals as follows:

Such radicals can recombine in the absence of oxygen. In the presence of oxygen peroxy radicals may be formed, which leads to the degradation of polymeric chains Potts (1991).

### Photodegradation

Photodegradation is degradation of a photodegradable molecule caused by the absorption of photons, particularly those wavelengths found in sunlight, such as infrared radiation, visible light, and ultraviolet light. However, other forms of electromagnetic radiation can cause photodegradation. Photodegradation includes photodissociation, the breakup of molecules into smaller pieces by photons. It also includes the change of a molecule's shape to make it irreversibly altered, such as the denaturing of proteins, and the addition of other atoms or molecules. A common photodegradation reaction is oxidation. Photodegradation in the environment is part of the process by which ambergris evolves from its fatty precursor. Photodegradation also destroys paintings and other artifacts.

Light - induced polymer degradation, or photodegradation, includes the physical and chemical changes caused by irradiation of polymers with ultraviolet or visible light. In order to be effective, light must be absorbed by the substrate (polymeric system). Thus, the existence of chromophoric groups in the macromolecules is a prerequisite for the initiation of any photochemical reaction Schnabel (1981).

Ketones, quinines, and peroxides are initiators for different reaction degradation or chemical modification occurring in organic compounds Kaczmarek et al. (1999). They absorb light up to about 380 nm, which causes their excitation or cleavage to radicals. One may initiate polymer degradation and other transformation by abstruction of hydrogen atom from a macromolecule (PH) and formation of polymer alkyl radical (P^.^) (Rabek 1993; Rabek 1996).

The influence of low-molecular weight organic compounds such as benzophenone (BPh), anthraquinone (AQ) and benzoyl peroxide (BPo) on the photoprocesses of polystyrene has been studied. The results indicate that additives accelerate and increase the photodegradation and photooxidation of polystyrene Kaczmarek et al. (1999).

Photodegradation may occur in the absence of oxygen (chain breaking or cross-linking) and the presence of oxygen (photooxidative) degradation. The photooxidative degradation process is induced by UV radiation and other catalysts (or both) and can be accelerated at elevated temperatures.

Photodegradation of polystyrene (e.g. embrittlement and color change) can take place upon irradiation with a portion of UV light that is contained within sun light.

Many reviews have been published on photodegradation and photooxidation degradation of polystyrene (Pinto et al. 2013; Gijsman and Diepens 2009; Rabek 1995).

The term of photodegradation might be distinguished from photooxidation of the polymer. In the latter, oxygen is involved in the process while in the former light energy (E=hƲ) only is responsible for the photodegradation.

### Mechanism of photooxidative degradation of polymers

Photooxidative degradation of polymers, which include processes such as chain scission, crosslinking and secondary oxidative reactions, and takes place via radical processes, similar to thermal oxidation reactions (Ranby and Rabek 1981; Carlsson *et al.*^1976^; Mc Kellar & Allen 1979; Rnby and Lucki 1980).

Two mechanisms have been proposed to explain the photooxidation of polymers in conformity with similar observations made on low molecular weight compounds. One proceeds through direct reaction of singlet oxygen with the substrate while the other involves the production of radicals and subsequent reaction with oxygen (Geuskens & David [Bibr CR39][Bibr CR40]; Trozzolo and Winslow 1968).

### (I)The singlet oxygen mechanism of oxidation

It has been clearly demonstrated that many photosensitized oxidation reactions proceed with participation of oxygen in an electronically excited singlet state. The photochemical production of singlet oxygen is mainly due to quenching of the excited triplet state of suitable sensitizers:

Singlet oxygen exhibits several specific reactions and the one that has been most often invoked in the photooxidation of polymers is the formation of a hydroperoxide by oxidation of an olefin containing an allylic hydrogen, and which could further decompose and lead to chain scission and formation of a terminal of carbonyl group.

### (II)The free radical mechanism of oxidation

The radical mechanism of photooxidation of polymers proceeds through a chain reaction similar to that for homogeneous liquid phase oxidations Kochi and Wiley (1973), Geuskens & David ([Bibr CR39][Bibr CR40]).

### Steps of photodegradation

Photooxidative degradation includes the following steps: (Rabek 1994; Rabek 1974; Guillet [Bibr CR50][Bibr CR51]).

Initiation, propagation and termination.

**Figure 2:**
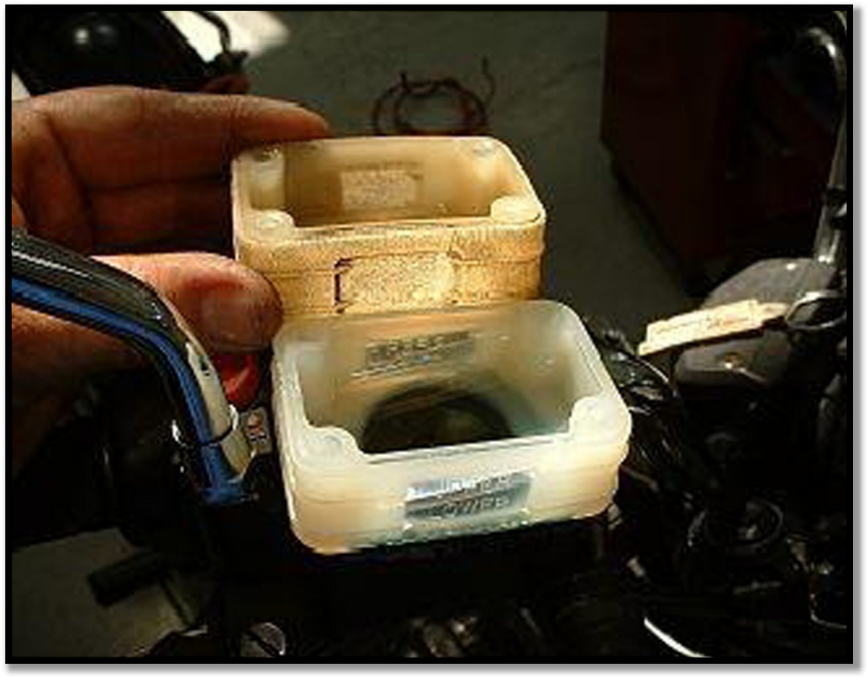
**A plastic item with thirty years of exposure to heat and cold, brake fluid, and sunlight.**

These steps are illustrated in the Figure 2:

### Photoinitiation step

Internal and/or external chromophoric groups absorb light and produce low molecular weight radicals (R^.^) and /or polymeric macro radicals (P^.^) as follows:

This reaction may be initiated by physical factors such as: UV radiation, heat, ionization, ultrasonic or mechanical effects or by chemical factors (direct reaction with O_2_ or atomic oxygen, catalysis or singlet O_2_ [O_2_^*^]^1^ excited state.

The “hydroperoxide” (POOH) is the most important initiator in the photooxidative process Grassie and Scott (1985).

Whatever the initial mechanism of radical formation, hydroperoxides are produced after reaction with oxygen. These are thus key intermediates in the oxidation of polymers Amin et al. (1975). Moreover, hydroperoxides are extremely photolabile; they usually decompose with quantum yields close to unity to produce radicals that can abstract hydrogen atoms from the polymer and thus initiate the photooxidation:

The formation of hydroperoxide and its photolysis illustrate the formation of other effective functional groups such as carbonyl during β-scission of the alkoxy radical Grassie and Scott (1985).

**Figure 3:**
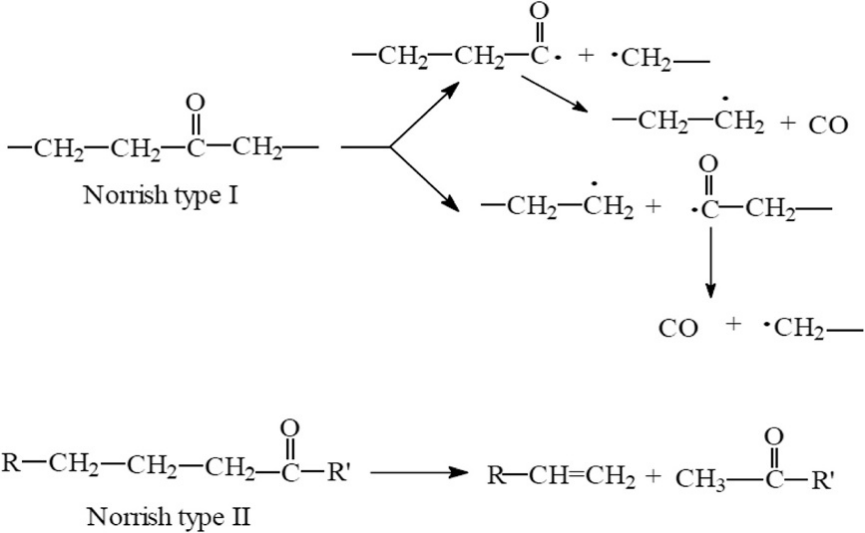
**The β scission in polymer molecule, Grassie and Scott (** 1985 **).**

**Figure 4:**
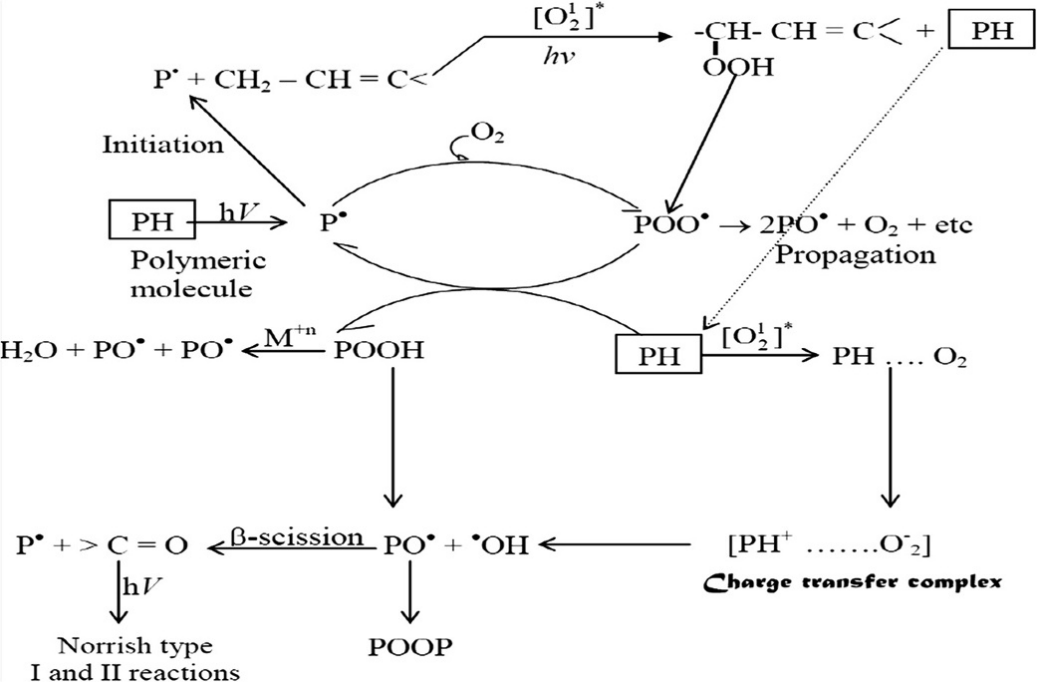
**Polymer photooxidation, Yousif (** 2012 **).**

In addition to the polymer oxidation initiation by the photolysis of hydroperoxide groups, a second major contributor to the photodegradation of polymers is ketone photolysis, which proceeds through two major reactions called Norrish I (radical generation and no chain cleavage) and Norrish II (chain cleavage), as shown below. Ketones are introduced onto the backbones of polymers by photooxidation. On exposure to light, these ketone groups absorb photons of appropriate energy, break carbon–carbon bonds, and scission the polymer backbone (Rabek 1995; Feldman 2002) as shown in the Figures 3 and 4 (Grassie and Scott 1985):

### Propagation step

This step can be divided into Rabek (1987) six different steps:

A) Subsequent reaction, of low molecular radicals (R^.^) and polymer alkyl radical (P^.^) in a chain process similar to the abstraction of hydrogen from the polymer molecule:
B) Reactions of polymer macro radicals with oxygen, during which polymer peroxy radicals (POO^.^) are formed.
C) Abstraction of hydrogen from the same or another polymer molecule by polymer alkylperoxy radical, with the formation of a hydroperoxide group.
D) Photodecomposition of hydroperoxide groups with the formation of polymer alkyloxy (PO^.^), polymer peroxy (POO^.^), and hydroxyl, (HO^.^) radicals.
E) Abstraction of hydrogen from the same or another polymer molecule by polymer alkyloxy radical with the formation of hydroxyl function groups in polymer.
F) Dispropertionation reaction (scission process) of polymer alkoxy radicals with the formation of aldehyde end groups and end polymer alkyl radicals.

There are many points concerning the mentioned propagation steps which are:

- Formation of propagating radicals (POO^.^) by reaction (8), in a semi-rigid polymer system is very low (ca.1%) Mayo (1978) because of efficient polymer alkyl radicals (P^.^) recombination before O_2_ react with them. Polymer alkylperoxy radicals (POO^.^) are strongly resonance stabilized, and they are stable at room temperature in polymer matrix. They are selective electrophilic species abstracting 3° hydrogen in preference to 2° or 1° (Mill 1978; Guillet 1978Yousif et al. 2011b).
- The light quanta produced by sun irradiation are energetically sufficient to cleave PO – OH and also P – OOH , but hardly POO – H bonds , which have the dissociation energies of 42 kcal/mol (PO – OH), 70 K cal/mol (P – OOH) , and 90 kcal/mol. (POO – H) Ranby and Rabek (1975b).The large differences in bond dissociation energy between PO – OH and P – OOH means that reaction with formation PO and OH radicals will predominate during light irradiation.
- The most probable mechanism of photo-decomposition of -OOH group occurs through energy transfer processes from the excited carbonyl or aromatic hydrocarbon groups (donors) to hydroperoxy groups (acceptors).

**Figure 5:**
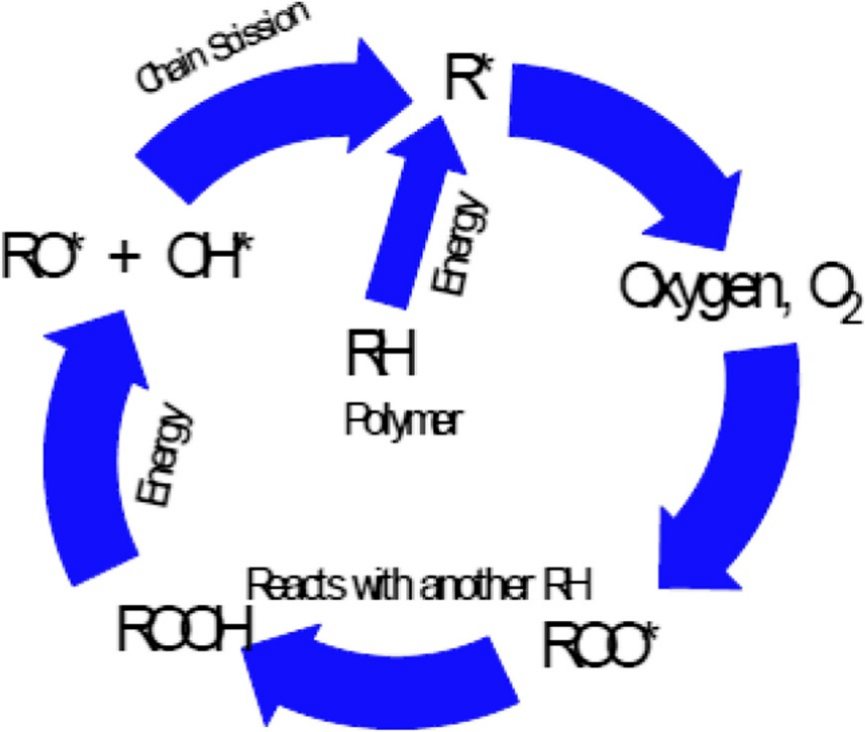
**Photooxidation cycle for polymers.**

Generally, the overall processes of photooxidative degradation are given in the Figure 4 Yousif (2012) (Figure 5):

### Termination step

**Figure 6:**
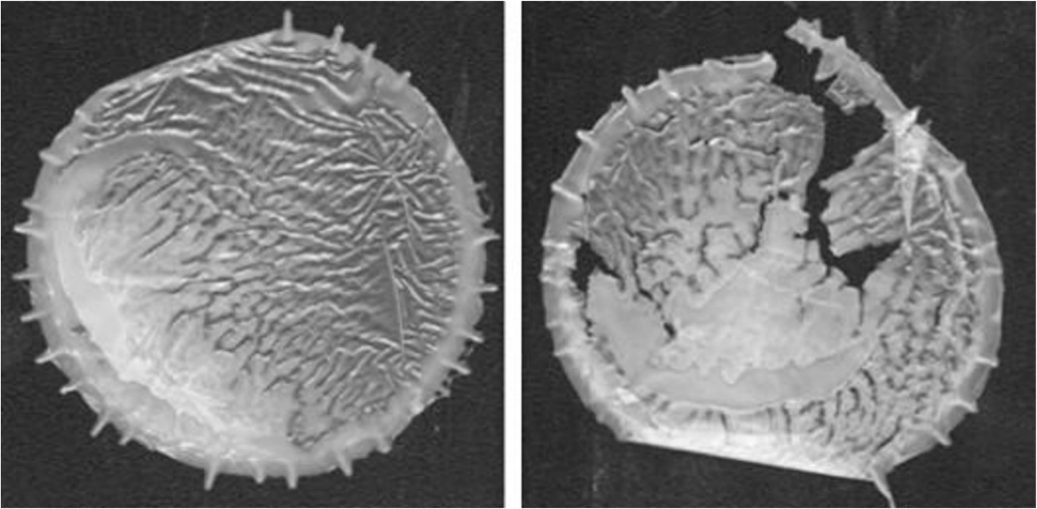
**Polymer film before and after exposure to UV light.**

The radicals formed in the degradation of polymers can be terminated by numerous different combination reactions between two polymer radicals, in which inactive products are formed:

When the oxygen pressure is high, the termination reaction follows reaction (17) almost fully. At low oxygen pressure other termination reactions take place to some extent. In the degradation of solid polymers when sufficient oxygen content cannot be maintained reaction (18) becomes significant. Polymer radicals can couple mutually as in reaction (19) and form crosslinks with polymer peroxy radicals (Rabek and Ranby 1975; Ruoko, 2012) (Figures 3 and 6).

### Polystyrene

Polystyrene (PS) is a multipurpose polymer that is used in varied applications in rigid and foamed form. General purpose polystyrene is clear and hard which is used in packaging, laboratory ware, and electronics Meenakshi et al. (2002).

### History of polystyrene

Edward Simon accidently discovered polystyrene in 1839 in Germany. From the resin of Turkish sweetgum tree, Liquidambar orientalis he obtained an oily substance, which thickened into jelly in air; he described it as styrol oxide. In 1845 John Blyth and August Wilhelm von Hofmann observed that the same changes occur in styrol in the absence of oxygen. They named it as metastyrol. Marcelin Berthelot in 1866 found that a polymerization process is responsible for the change styrol to metastyrol. In the thesis of Hermann Staudinger (1881–1965) it was reported that a chain reaction occurs in styrol resulting in the formation of macromolecules which was later called polystyrene. In Germany, I. G. Farben, company in 1931 started producing polystyrene in Ludwigshafen. The Koppers Company in Pittsburgh, Pennsylvania produced expanded polystyrene in 1959.

### Synthesis of polystyrene

**Figure 7:**
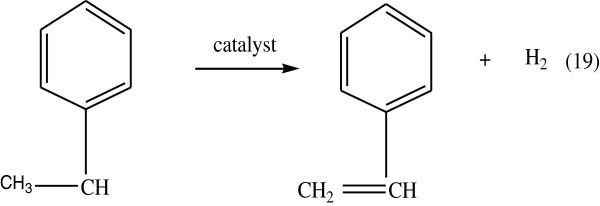
**Synthesis of styrene.**

Polystyrene is manufactured by the addition polymerization of the styrene monomer unit. At room temperature, polystyrene is normally a solid thermoplastic, but can be melted at higher temperature for molding or extrusion, then resolidified. Styrene is an aromatic monomer, and polystyrene is an aromatic polymer (Ruoko, 2012). The main manufacturing route to styrene is the direct catalytic dehydrogenation of ethyl benzene as in Figure 7.

The reaction shown above has a heat of reaction of -121 Kj/mol (endothermic). Nearly 65% of all styrene is used to produce polystyrene.

**Figure 8:**
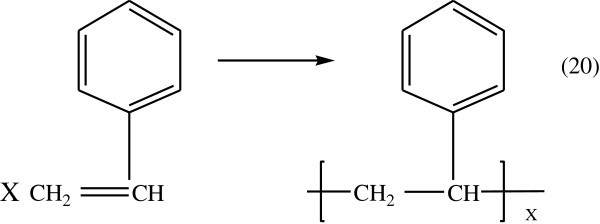
**Polymerization of styrene.**

The overall reaction describing the styrene polymerization was shown in Figure 8.

Polystyrene is produced radical- initiated suspension polymerization. And some is produced by reactive extraction.

### Types of polystyrene

**Figure 9:**
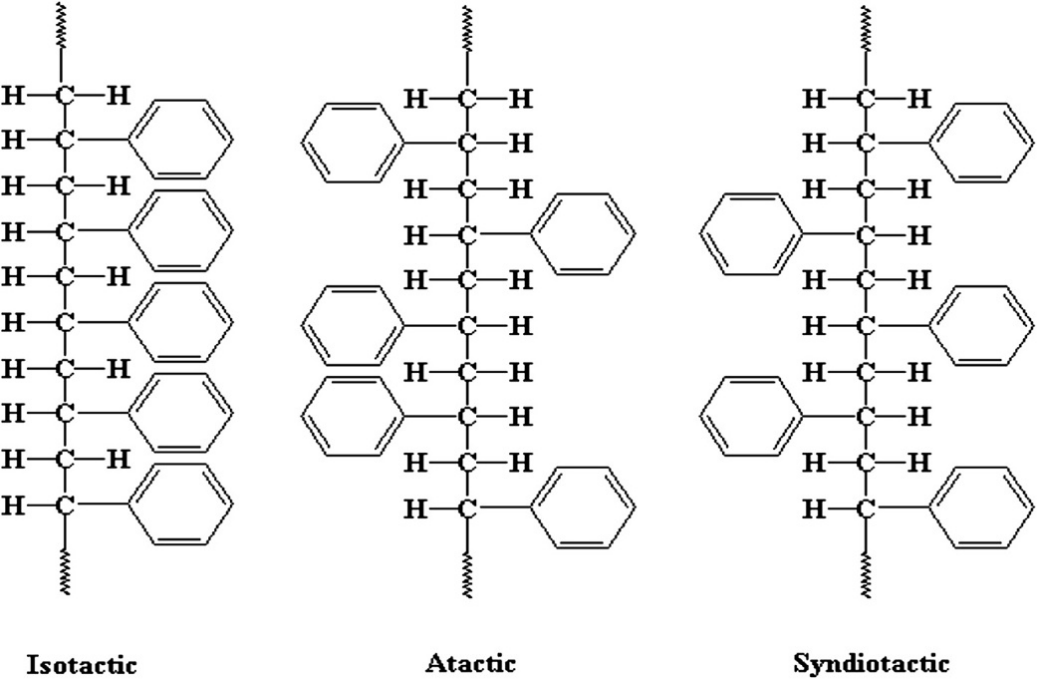
**Types of polystyrene.**

On the basis of structure polystyrene can be classified into three forms Figure 6. The polystyrene containing all of the phenyl groups on one side is of the planer zig – zag mechanism termed as isotactic polystyrene. If the phenyl groups are randomly distributed then it is called atactic polystyrene. Radical vinyl polymerization yields atactic polystyrene. The polystyrene containing phenyl groups on alternating sides of the chain is described as syndiotactic polystyrene (sPS), which is highly crystalline. It has the tendency to crystallize very quickly which gives it the favourable properties of high melting temperature and chemical resistance (Saitoh et al. 2003; Gupper and Kazarian 2005) (Figure 9).

### Uses of polystyrene

Polystyrene is a type of plastic that is used for a variety of functions including in rigid items such as refrigerator crispers, coat hangers, DVD cases and printer cartridges. One of the important uses of PS is in the manufacture of cover signal lamps of some automobiles Safy Eldin and El-laithy (1994).

It is a brittle, high glass transition temperature polymer. Pure solid polystyrene is a colorless, hard plastic with limited flexibility which can be cast into molds with fine detail. Polystyrene is used in numerous plastic products. It is economical and is used for producing plastic model assembly kits, plastic cutlery, Fried (2003) for packaging and in sulation materials in the form of a foam or bead, and in electronics, construction, house and medical ware and disposable food services Meenakshi et al. (2002) and many other objects where a fairly rigid, economical plastic of any of various colors is desired.

Polystyrene fabricated into a sheet can be stamped (formed) into economic, disposable cups, glasses, bowls, lids, and other items, especially when high strength, durability, and heat resistance are not essential. A thin layer of transparent polystyrene treditimatly been used as an infrared spectroscopy standard. This is the lightweight material of which coffee cups and takeaway food containers are made. The voids filled with trapped air give expanded polystyrene low thermal conductivity. It is also used as insulation in the wall of building.

When a polymer matrix is mixed with inorganic nanoparticles, the thermal (Garcia et al. 2009), mechanical (Uhl et al. 2002), optical, electrical, magnetic and flammability properties of such a nanocomposite are much different from the polymer matrix itself Manzi-Nshuti et al. (2009).

### Environmental effects of polystyrene

Synthetic plastics are used in many fields such as packing, household, agricultural, marine and architectural. Plastics have replaced natural resources such as cotton, wood and metals because of their light-weight, and durability. PS is a widely used thermoplastic. Its hardness, hydrophobic nature and chemical composition cause it to persist in nature without any decomposition for long period of time thus cause environmental pollution Singh and Sharma (2008). As waste plastic material has become a serious problem, recycling is receiving attention as a means of preserving the environment and reserving resources Pantano et al. (2009). Polystyrene waste requires the transportation of big large volume of materials, which is costly and makes recycling economically unfeasible.

### Degradation of polystyrene

Polymers are weathered due to environmental factors such as light and temperature. The conditions of use play a key role in the degradation of plastics. Polystyrene losses its mechanical and tensile properties due to effect of UV light and heat Kiatkamjornwong et al. (1999). UV light induces the production of radicals by oxidation. Radicals cause the chains of polymer to breakdown.

### Consumption of polystyrene in the world

**Figure 10:**
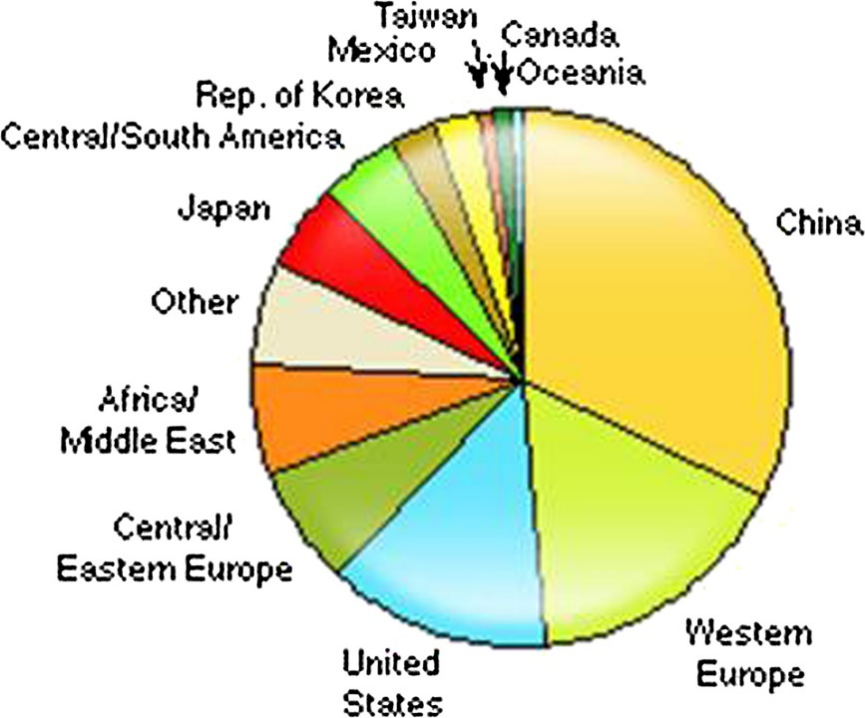
**World consumption of polystyrene–2010.**

Polystyrene, a thermoplastic resin that is easily processed has many applications such as disposables, packaging, toys, construction, electronics and houseware. The following pie chart shows world consumption of polystyrene (see Figure 10).

Asia is the overall leader in production and consumption of polystyrene, with 53% of total world production and 47% of total consumption of polystyrene in 2010. North America and Western Europe follow distantly at about 17-19% of the total production and consumption each. Through the recession, the percentage of imports of EPS resin relative to consumption rose from 40% to 44% over 2007 through 2010. Global demand for polystyrene and expandable polystyrene increased from 13 mln tons in 2000 to around 14.9 mln tons in 2010. Packaging, construction, appliances and consumer electronics industries were the major consumers of polystyrene and expandable polystyrene.

The global demand for polystyrene and EPS is rising within developing countries such as China, India, Iran, Saudi Arabia and Brazil. Slovakia’s EPS consumption follows the trend set by other EU countries, whose year-on-year increase in consumption was about 3.3 percent during the monitored period. Of all EU countries, France, Poland, Austria and Germany reported the highest consumption of EPS. The lowest consumption was registered in Greece, Hungary, Italy and Spain. Complete insulation of a building using EPS can achieve a reduction of up to 66 percent in the heat consumption of prefabricated buildings, while in the case of detached family houses this can rise to as much as 70–79 percent. The life span of such insulation is about 50 years.

### Photodegradation of polystyrene

Photodegradation of polystyrene in air causes discoloration (yellowing), cross-linking, and chain scission due to oxidation. A photodegradation process was also proposed on the basis of the IR spectrum of the photoirradiated film, which indicated the formation of peroxy radical and hydroperoxide intermediates. The photochemical reactions cause the dissociation of a polystyryl radical by creating an electrochemical excited state. The polystyryl radical is converted to peroxy radicals by reacting with oxygen. The peroxy radical undergoes chain scission and formation of carbonyl compounds.

**Figure 11:**
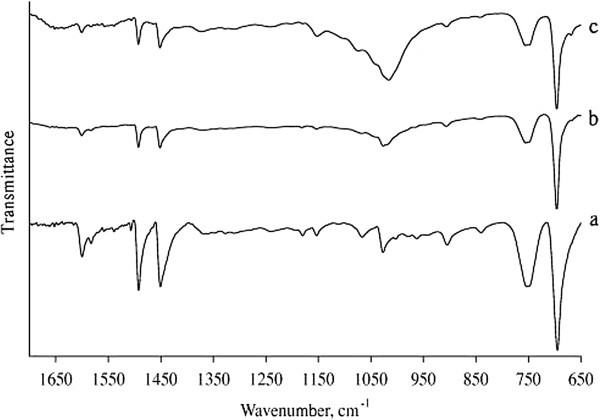
**FTIR spectra of (a) pure polystyrene and after (b) 30 days and (c) 15 days of degradation, (Pushpadass et al.** 2010 **).**

**Figure 12:**
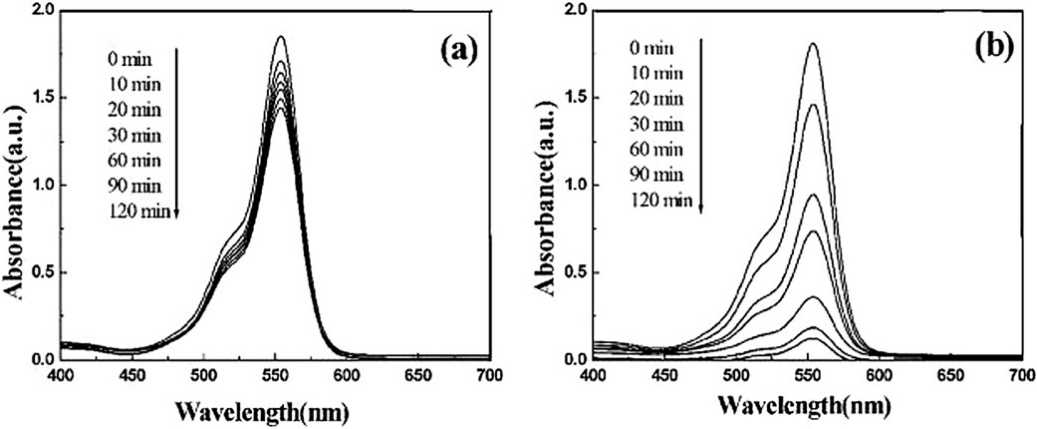
**Change in UV. Vis. spectram of polystyrene film after irradiation time, Shao et al. (** 2013 **).**

The photosensitivity has been evaluated of plastic sheet after different exposure times (0.5, 5, 10 or 21 hr.). FT-IR analyses of photo irradiated polystyrene showed increase in absorption peaks at 1742-1745cm^-1^ indicating the presence of ketone carbonyl groups. For polymer of hydrocarbon, oxidation must precede biodegradation. Kiatkamjornwong et al. (1999) (see Figures 11 and 12).

### Photooxidative degradation of polystyrene by radical processes

All commercial organic polymers degrade in air when exposed to sunlight as the energy of sunlight is sufficient to cause the breakdown of polymeric C–C bonds as a consequence of degradation. The resulting smaller fragments do not contribute effectively to the mechanical properties and the polymeric article become brittle. Thus the life of thermoplastics for outdoor applications becomes limited due to weathering Andrady *et al*. (1998).

When polystyrene is subjected to UV irradiation in the presence of air, it undergoes a rapid yellowing and a gradual embrittlement. Industrially, the yellow discoloration is an important adverse effect of the ageing of polystyrene, e.g. during exposure outdoors (Geuskens et al. [Bibr CR41][Bibr CR42]; Weir 1978; Lucki and Rnby 1979; Rnby and Lucki 1980).

Photodegradation and photooxidation of polystyrene film and solutions have been widely investigated (Rabek & Ranby [Bibr CR90][Bibr CR91]; Lawrence and Weir 1973).

The main reactions observed are bond session, chain crosslinking and oxidative degradation. In spite of these great efforts the mode of photodegradation of polystyrene is still very controversial. A number of impurities and irregularities in polystyrene, such as hydroperoxides, aromatic carbonyl groups, olefin bonds and chain peroxide linkages, can be responsible for the photoinitiation of the radical oxidation of polystyrene Rabek (1987).

Irradiation of polystyrene by UV. and visible light with wavelength (λ< 400) nm., leads to the formation of radicals whose nature is dependent on the spectral composition of the light and irradiation condition Kuzina and Mikhailov (1993). The end group of PS or ketonic impurities also absorb the light of wave lengths longer than 300 nm (Geuskens and David [Bibr CR39][Bibr CR40]; Klopffer 1975; George and Hodgeman 1977).

**Figure 13:**
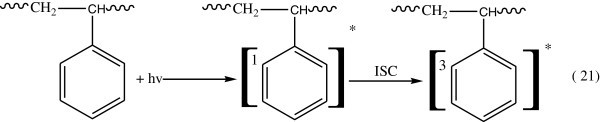
**Formation of triplet state after absorption of light by benzene ring in PS.**

Absorption of light quanta by benzene rings is the first step producing excitation of the rings at excited singlet states which are transformed by intersystem crossing (ISC) to the triplet sate as Figure 13.

**Figure 14:**
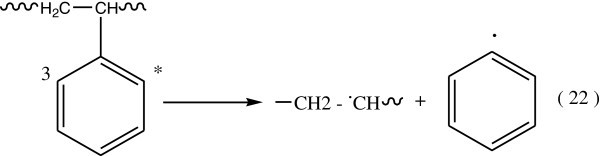
**Dissociation of C6H5-C bond.**

**Figure 15:**
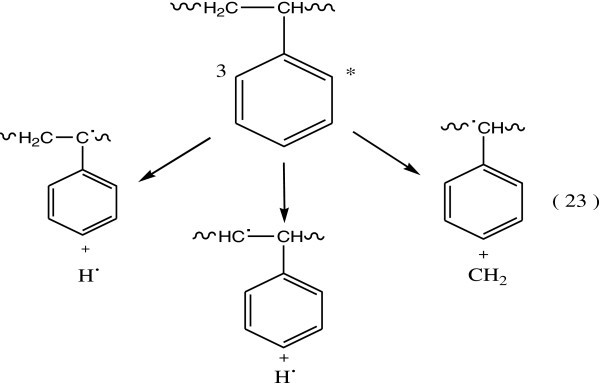
**Formation of C-H and C-C bonds.**

The secondary step in the reaction of triplet state of benzene, which occurs by one of the following two modes:

1. Triplet energy of the excited benzene may be give rise to the dissociation of the C6H5 – C bond (Figure 14):
2. Triplet energy excitation can be transferred by intramolecular energy transfer processes to the C – H or C – C bonds (Figure 15).

It has been suggested that the most important step in photolysis of polystyrene in the absence of oxygen is the scission of a C – H bond. The electron spin resonance (ESR) spectra obtained during the UV, irradiation of polystyrene (PS) were interpreted as arise from radicals formed in reaction (8) Cozzen and Fox (1969); Grassie and Weir (1965). The hydrogen radicals are very mobile; they can diffuse out from the polymer matrix and then recombine with each other to give molecular hydrogen.

The generally accepted theories of oxidation of polymers are based on the radical process for the thermal oxidation of hydrocarbons. This process involves peroxy group formation and it proceeds through the following fundamental steps Ranby and Rabek (1975a): initiation, propagation, radical chain branching and termination.

#### Initiation

In each repeating unit of polystyrene there is a phenyl chromophore that shifts the absorption spectrum of PS towards the visible region, up to λ≈260 nm. When PS is irradiated with UV radiation the phenyl ring is excited, after which the excitation energy is transferred to the nearest C-H bond, resulting in the cleavage of hydrogen and leading to polymer radical formation:

The type of the radicals formed during polystyrene photodegradation depends on the irradiation wavelength Ranby and Rabek (1975).

**Figure 16:**
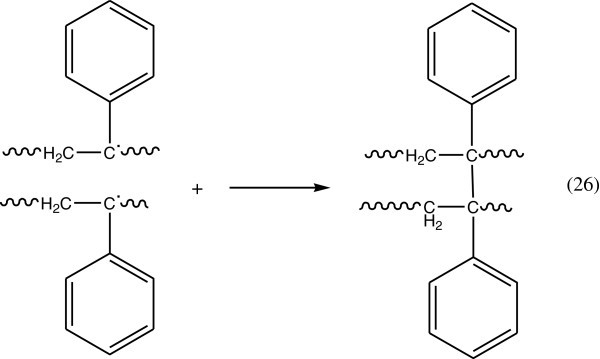
**Formation of cross-linking when two macroradical are near.**

The movement of polymer macromolecules in the solid state is restricted, but radicals can migrate along the polymer chain until they trapped by other radicals or by impurities. When two macro radicals are near to each other cross-linking may occur as in Figure 16.

**Figure 17:**
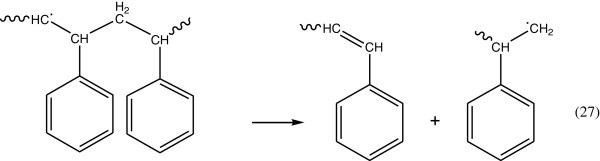
**Macroradical disproportionate.**

On the other hand, macroradicals may disproportionate leading to chain scission (Figure 17).

**Figure 18:**
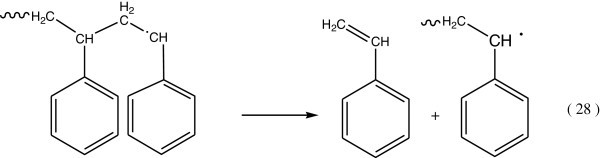
**Formation of Styrene monomer.**

When an end group radical is formed, unzipping may occur with the formation of monomer see Figure 18.

**Figure 19:**
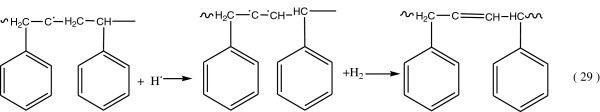
**Built up of conjugated bond sequences in the polybachbone.**

During UV irradiation PS samples show increased optical density and slight yellowing. This coloration of PS has been attributed. Grassie and Weir (1965) to the build -UP of conjugated bond sequences in the polymer backbone (Figure 19).

#### Propagation reaction

**Figure 20:**
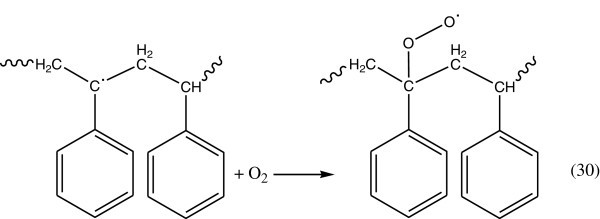
**Producing peroxy radical.**

Polystyrene alkyl radicals (R^.^) can easily react with molecular oxygen (triplet state), to produce peroxy radicals by addition (Figure 20). Baloglu and Fisch (1994).

**Figure 21:**
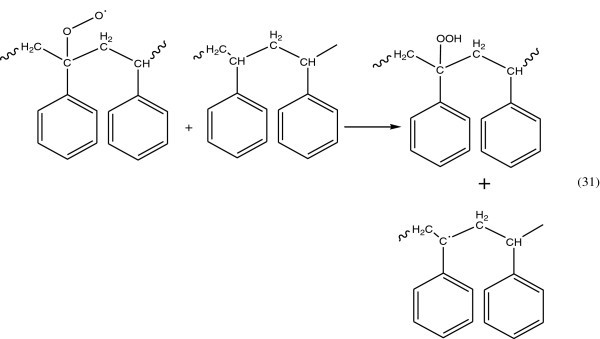
**Polymer peroxy radical abstracted hydrogen.**

A polymer peroxy radical can react with the surrounding polystyrene molecule and abstract hydrogen atom see Figure 21.

**Figure 22:**
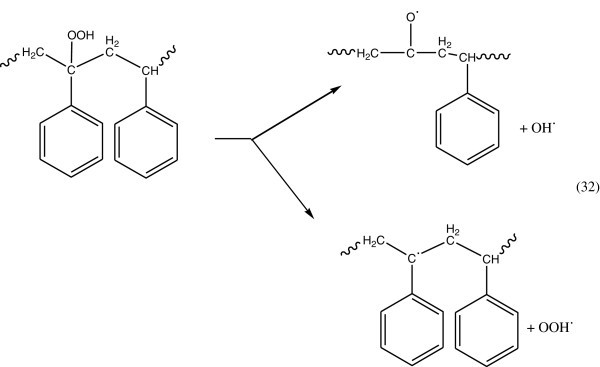
**Decomposition of hydroperoxide.**

On exposure to light the polymer hydroperoxides can decompose according to the following reactions as shown in Figure 22.

**Figure 23:**
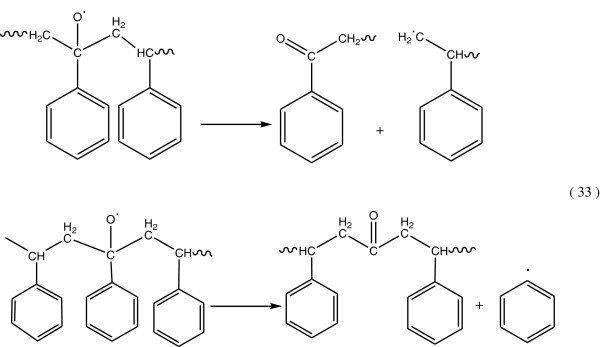
**Formation of alkoxy polymer radicals.**

Alkoxy polymer radicals produced during the photolysis of hydroperoxide groups may decompose by β-scission reaction and to form a cetophenone and chain aliphatic ketone (Figure 23) (Kuzina and Mikhailov 1998; Geuskens et al. [Bibr CR41][Bibr CR42]).

**Figure 24:**
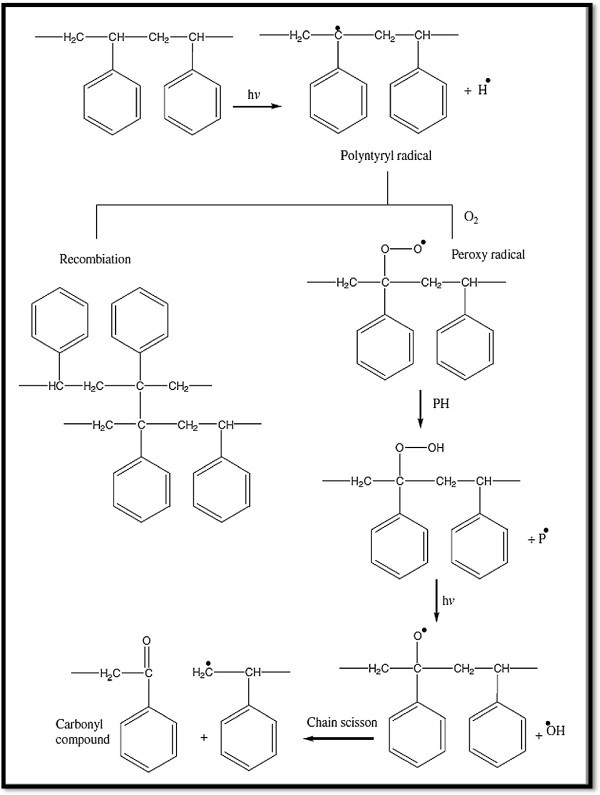
**General mechanism of photodegradation of polystyrene, Yousif et al. (** 2011a 2011b **).**

The general process for photodegradation of polystyrene was shown in Figure 24, (Yousif *et al.*, 2011a).

### Photostabilization mechanisms in polymers

It is well known that all commonly used plastics degrade under the influence of sunlight. Almost all synthetic polymers require stabilization against the adverse effects; with the development of synthetic resins it became necessary to find ways and means to prevent, or at least reduce, the damage caused by the environmental parameters such as light, air and heat. That is why the photostability of polymers is one of the most important considerations for application.

Photostability can be achieved through the addition of special chemicals, light stabilizers or UV stabilizers, that have to be adjusted to the nature of the resin and the specific application considered.

The photostabilization of polymers involves the retardation or elimination of photochemical process in polymers and plastics that occur during irradiation. There are several different methods of photostabilization, and the choice of method is dependent on the polymer and application.The most important methods of photostabilization are the screening or absorbing of UV radiation by stabilizing agents and the use of antioxidants, which react with the polymer radicals, stopping the degradation process by forming inactive products (Ruoko 2012; Yousif *et al.*[Bibr CR128][Bibr CR129]).

The following stabilizing systems have been developed which depend on the action of stabilizer: (a) light screeners, (b) UV absorbers, (c) excited state quenchers, (d) peroxide decomposers and (e) radical scavengers. Of these, it is generally believed that types c, d and e are the most effective Yousif et al. ([Bibr CR128][Bibr CR129]).

Practice shows that when the polymer contains a photostabilizer, the oxidation rate is much reduced. Stabilizers reduce but do not completely prevent oxidation, so it can be expected that some reaction will take place in the interior of a stabilized polymer.

### Ultraviolet Stabilizers

Ultraviolet Stabilizers additives for plastics and other polymer materials which prevent the photodegradation or photocrosslinking caused by ultraviolet light.

The UV absorber and screener operate by absorbing the incident UV radiation preventing it from reaching the bulk of the polymer and converting the energy thus acquired into a less damaging form such as heat or they may act as radiation reflecting or scattering (preferably coating) Decker and Biry (1996) on the surface of the polymer thus delaying discoloration and delamination.

An important disadvantage of UV absorbers is the fact that they need a certain absorption depth (item thickness) to provide good protection to a polymer. Therefore, the protection of polymer surfaces and of thin items such as films or fibers is only moderate Gugumus (1987).

The amount of an absorber required to provide economical protection in a plastic is governed by several **factors** such as:

i. Thickness of the plastic.
ii. Tolerance of color.
iii. Effect of high concentration of absorber in plastics.
iv. Compatibility of the absorber in the plastic.

The most common UV stabilizers are: UV absorber and light screeners, quenchers, hydroperoxide decomposers, radical scavengers and singlet oxygen, (^1^O_2_).

**Ultraviolet stabilizers can be classified** according to their mechanisms of action in the photostabilization process into:

(i) UV absorber and light screeners.
(ii) Quenchers.
(iii) Hydroperoxide decomposers.
(iv) Radical scavengers.
(v) Singlet oxygen, (^1^O_2_), quenchers.

### Rabek (**The UV stabilizer can be classified into:**^1987^)

(i) Pigments.
(ii) Metal chelates.
(iii) Carbon black.
(iv) Salicylates.
(v) Salicylanilides.
(vi) Hydroxy phenyl benzotriazoles.
(vii) Hydroxy benzophenones.

### Mode of action of UV. Absorber

The action of UV absorber is relatively simple, it interact with the first step of the photooxidation process, that is, it absorbs the harmful UV radiation before it reaches to the photo active chromophoric species in the polymer molecule (300–400) nm. Thus the energy dissipates in a manner that does not lead to photosensitization. A UV absorber must be light stable, because otherwise it would be destroyed in stabilizing reactions.

Certain phenolic and non-phenolic UV absorbers with structures (1), (2), (3) and (4) respectively, display high performance and inherent photostability in the (300–400) nm. region required for protection of polymers against photodegradation .The structure of some UV absorbers are shown in Figure 6: Pospisil and Nespurek (2000).

**Figure 25:**
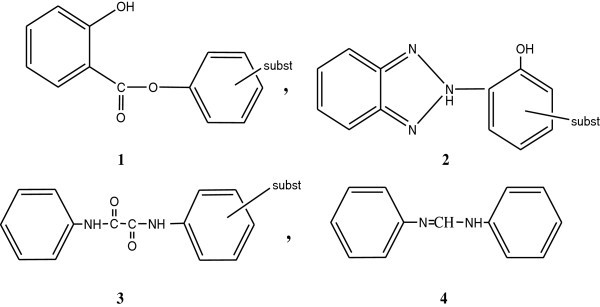
**Examples of UV absorbers.**

Hydroxy benzophenones and hydroxyphenyl bezotriazoles are the most extensively studied UV absorbers Gugumus (1979) (see Figure 25).

Generally, after photons are absorbed by the chromophores, molecules rise to higher excited state, by spin inversion of singlet state. The singlet states may change to triplet state by intersystem crossing (ISC) or return to a lower excited state by internal conversion (IC) (these are radiationless processes). Excited states have the ability to lose the excitation energy by some radiation processes such as fluorescence or phosphorescence or by energy transfer to another molecule.

Concerning the energy transformation in hydroxy benzophenones, it has been shown with 2-hydroxy benzophenone (Gugumus 1979; Allen 1983), that on exposure to light, it is transformed into the enol form which turns back into its initial form on losing thermal energy to the medium. On the basis of spectroscopic data, it is concluded that the energy transformation involves exclusively a fast radiationless transition.The photostabilizing process of *O*-hydroxy benzophenone has been the subject of many investigations Collell & Amer (1968). The most probable process involved is the utilization of the energy of the absorbed photon to intramolecular proton transfer during which a quinone structure is formed.

**Figure 26:**
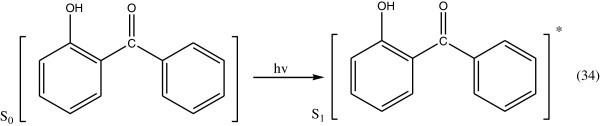
***O-*** **hydroxybenzophenone absorbed a photon.**

An *O*-hydroxyphenone molecule in the ground state (S_0_) absorbs a photon as is excited to the singlet state (S_1_) (as in Figure 26), Yousif (2012).

**Figure 27:**
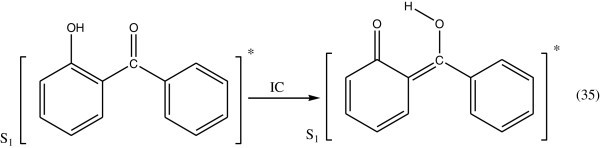
**Formation of Quinone.**

In this excited singlet state (S_1_) the proton of a hydroxyl group is transferred to the carbonyl group during which a quinone structure is formed (Figure 27).

**Figure 28:**
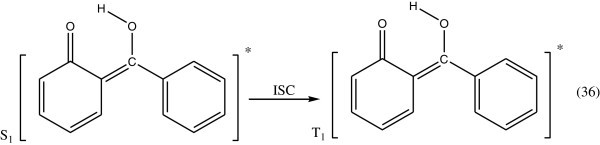
**Formation of excited triplate state of** ***O-*** **hydroxybenzophenone.**

The excited singlet sate (S_1_) of the quinone structure is converted by intersystem crossing (ISC) to the excited triplet state (T_1_) (Figure 28).

**Figure 29:**
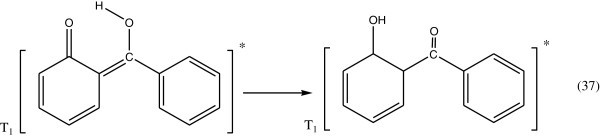
**Stable structure of excited triplate state of** ***O-*** **hydroxybenzophenone.**

Since in the excited triplet state (T1), the stable structure is the ketone structure, see Figure 29.

**Figure 30:**
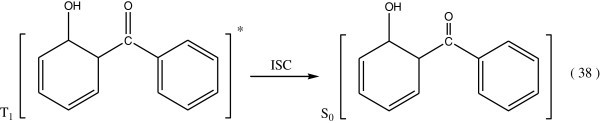
**Formation of singlate excited state of** ***O-*** **hydroxybenzophenone.**

The intersystem crossing (ISC) process leads from the excited triplet sate (T_1_) of the ketone structure directly to the ground state (S_0_) (Figure 30).

It has been shown that urethane and silylated derivatives of hydroxybenzophenones are effective photostabilizers, but cannot photostabilize polymers by intramolecular proton transfer Olson and Schroeter (1978). These compounds can act as: (Senna et al. 2002; Pospisil et al. 2002; Patel, 2007).

(i) Light screeners.
(ii) Excited state quenchers of chromophoric groups present in polymers.
(iii) Radical scavengers.
(iv) Hydroperoxide decomposers.

The photostabilizing effect of many aromatic compounds to be UV stabilizer for several polymers has been reported. This is due to their filtrating action which depends in turn, on their absorption characteristics (Ranby and Rabek 1975; Heller 1969).

### Pigments

Pigments are insoluble inorganic or mineral and organic compounds of complex structure, such as:

Powder metal (aluminum) is excellent reflector to UV light.

Fe_2_O_3_, Fe_3_O_4_, ZnO,TiO_2_… are inorganic pigment excellent UV screener.

Organic pigments such as azo and anthraquenone display good UV light absorption.

Pigments used as additives, are incorporated into polymers, coating, inks, etc.…. for:

(i) Cost reduction.
(ii) Reinforcement.
(iii) Hardening.
(iv) Improving slip and skid resistance.
(v) Color effect.
(vi) Storage stability.

### A pigment as a light screener should be

(i) Light stable for long term performance without fading.
(ii) Heat stable to withstand polymer processing conditions.
(iii) Migration resistant.
(iv) Low cost and non-toxic.

The influence of pigments in polymer photostability is not completely understood. If an absorbing pigment is introduced into a polymer, it acts as an inner screen for photo products. If these products are not photooxidized, they accumulate in the polymer matrix. Since pigments act as highly absorbing additives, photooxidative phenomena will be limited mainly to the surface of samples Yousif (2012); Yousif (2010).

### Carbon black

**Figure 31:**
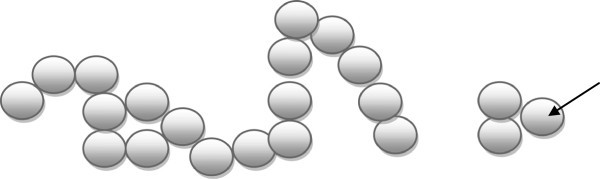
**The micro structure of carbon black particles, (Chirinos** 1989 **).**

Carbon black is one of the most efficient and widespread light absorbers. It consists of very fine particles fused together to form primary aggregates. The micro structure of carbon black particles is illustrated in Figure 31, David and Hsuan (2003).

Carbon black contains different functional groups such as carbonyl, hydroxyl, quinone, ether, etc.…

Carbon black absorbs UV radiation more efficiently than conventional colored pigments, carbon black are efficient light stabilizers for polymers Winslow et al. (1969) such as polyethylene. Their high efficiency as light stabilizers is probably due to their ability to act as inner filters for UV radiation, and as radical scavengers, because many of the carbon blacks contain stable radicals Szwarc (1956), and quenchers of singlet and triplet states of polymers. The effectiveness of carbon blacks is dependent upon Williams et al. (1969) the type of carbon black, the particle size and the degree of dispersion in the polymer phase.

Thus the UV stabilization efficiency of carbon black increases as the particle size decrease. An optimum concentration of carbon black is 3-5%. Higher than this concentration, the polymer looses it tensile strength and other mechanical properties David and Hsuan (2003).

### Quenchers

Quenchers deactivate excited states (singlet and/or triplet) of chromophoric groups in polymers before bond scission can occur by two mechanisms (Wiles 1979; Wiles and Carlsson 1980).

1. Energy transfer process.
2. Chemical and/or physical deactivation.

**Figure 32:**
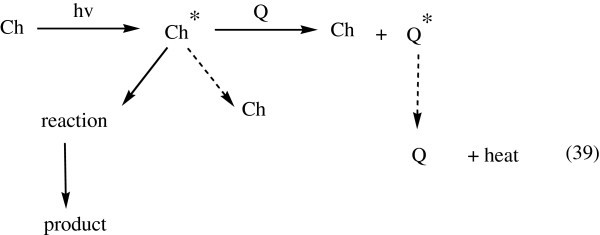
**General processes for Quenchers.**

The excited state of a chromophore (Ch) {where Ch is a chromophore such as dyes, pigments, antioxidant products, carbonyl groups, a variety of impurities or pollutants such as polynuclear aromatic compounds} may react but it can also be made to transfer its excess electronic energy to a quenching entity (Q) (see Figure 32).

If energy transfer to the quencher can compete with reaction, decomposition… etc. by Ch, and if Q can dissipate the excess energy harmlessly, then the system is stabilized. Energy transfer can occur efficiently only if the energy level of the quencher is below that of the chromophore Lamolla (1969).

Quenching of electronically excited states (singlets or triplets) is frequently described in terms of two distinct phenomena:

(i) Long range energy transfer, e.g., a dipole/dipole interaction as is considered to operate between the chromophore and quencher even at distaices >50A if there is significant overlap between the emission spectrum of Ch and the absorption spectrum of Q Lamolla (1969).
(ii) Contact (collisional) energy transfer of various kinds is said to require that Ch* and Q be within 10 to 15. In practice, the quenching of excited triplets is usually ascribed to collisional transfer (Beavan and Phillips 1974; Dan *et al.*^1973^).

In the solid state, transfer of energy occurs by conjugative or dipole-dipole interactions. The photooxidative degradation is promoted by the electronically excited oxygen molecule (singlet oxygen ^1^O_2_) formed in polymers. The [^1^O_2_] can also be generated in polymers by energy transfer from electronically excited carbonyl group to dissolved molecular oxygen Heskins and Guillet (1968).

The quenching of [^1^O_2_] is also necessary for effective stabilization. Nickel chelates have been proven to be very effective quenchers for the excited state of [^1^O_2_] Schmitt and Hirt (1963).

### Energy transfer

Energy transfer in a polymer is very important as this transfer is directly related to the effects of photostabilization and photodegradation Guillet ([Bibr CR50][Bibr CR51]).

The energy transfer in polymers can occur by one of the following processes:

(i) Intermolecular energy transfer

This process may occur between an excited polymer molecule (donor) and the molecule of a photostabilizer (acceptor) or between an excited sensitizer (donor) and a polymer molecule (acceptor).

(ii) Intramolecular energy transfer

This process occurs within same polymer molecule, e.g. between an excited chromophore in the chain (donor) and another segment of the polymer molecule (acceptor). This process is especially interesting in co-polymers. Guillet ([Bibr CR50][Bibr CR51]).

Nickel chelates are very effective quenchers of the triplet state of carbonyl groups in polyolefins. These chelates have been tested for photostabilization of polyisobutylene, poly butadiene Lala and Rabek (1980), polystyrene George (1974), PVC, poly (2,6-dimethyl-1,4-phenyloxide) and poly urethanes. (Chandra 1983; Osawa et al. 1979).

Nickel chelates can photostabilize a polymer by one or more of the following mechanisms.

(i) Quenching of the excited state of carbonyl groups (ketones) through energy transfer.
(ii) Quenching of the singlet oxygen (^1^O_2_) Allen et al. (1984).
(iii) Decomposion of the hydroperoxides (OOH) radical, to non radical inactive species Yousif (2010).
(iv) Scavenging of radicals (alkylperoxy (ROO^.^) and alkyloxy (RO^.^) Allen et al. (1982).

### Hydroperoxide decomposer

These compounds operate by reacting directly with polymeric hydroperoxide (ROOH). The decomposition of hydroperoxide in polymer to non radical derivatives was first demonstrated Carlsson and Wiles (1974). Many metal complexes of sulphur containing ligands such as dialkylthiocarbonate and dialkylthiophosphate not only decompose hydroxide in PE film but are also effective in UV stabilization (as UV absorbers and excited state quencher).

Generally there are three groups of compounds which operate by quite distinct process Aliwi (2001).

**Figure 33:**
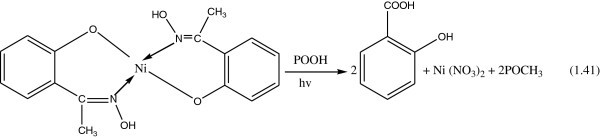
**Example of hydroperoxide decomposer.**

Compounds that belong to this type are organic phosphites and some Nickel chelates. They function by reducing hydroperoxide stochiometrically as presented in the Figure 33.

### Hindered amine peroxide decomposer

Hindered amine light stabilizers (HALS) have been found to be remarkably effective in performing both radical trapping and hydroperoxide decomposition.

These are one of the most effective photostabilizers for polymers and have been used in a large number of commercial polymers. They are efficient and cost-effective in many applications, despite their high prices Willis (1984).

The mechanism of (HALS) activity includes scavenging of R^.^, ROO and deactivation of hydroperoxides (ROOH) and peracids, which are based on a complex of chemical transformations. Nitroxide the key intermediate formed from (HALS), is prone to scavenge alkyl and form *O*-alkylhydroxylamines Bagheri *et al.* (1982). Therefore HALS of different structure are able to interconvert in cyclic pathways, destroying species which could lead to polymer degradation and coating species which protect the polymer against degradation Willis (1984).

**Figure 34:**
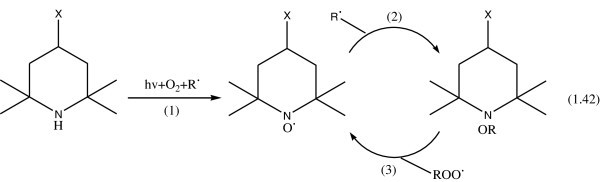
**The general cycle for protecting a polymer against degradation by HALS, Willis (** 1984 **).**

It has been generally accepted that hindered piperidine (e.g., 2,2,6,6- tetramethyl piperidine, which is the simplest model compound for HALS), during UV irradiation in the presence of oxygen (air) and radicals (R^.^), produces hindered piperidinoxy radicals according to the reaction 1 Yousif et al. (2011b): Piperindoxy radicals react with alkyl radicals (R^.^) to give a substituted hydroxypiperidine as shown in reaction 2 in Figure 34.

For the reaction of nitroxyl with (POOH) precedes via the formation of an intermediate (hydroxylamine) which reacts with (PO^.^) to generate the nitroxyl as reaction 3 as in Figure 31; Kurumoda et al. (1985).

Recently, different types of hindered amine light stabilizer have been synthesized to improve the compatibility of the additive with the polymer and enhance the long term of retention during the aging of polystyrene.

**Figure 35:**
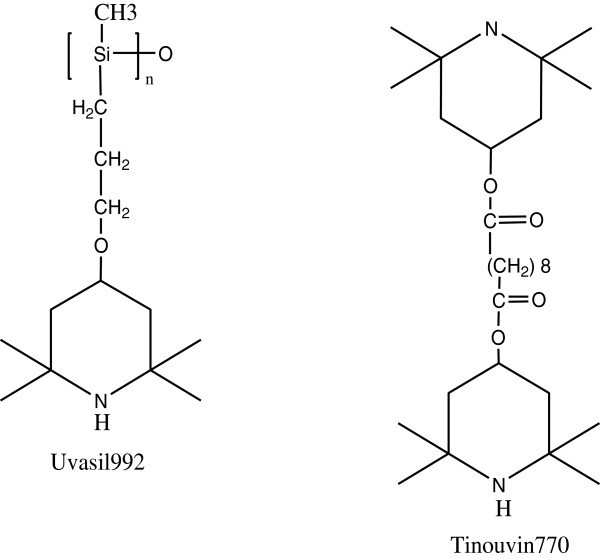
**The chemical structures of HALS, Bottino et al. (** 2004 **).**

HALS reduces the photooxidation by acting as radical scavengers. HALS structures are shown in the Figure 35 Bottino et al. (2004).

### Radical scavengers

The scavenging of radical intermediates is another possibility for polymer stabilization, analogous to that used in thermal degradation. Quinones react with alkyl radicals to form radicals that do not initiate polymer oxidation.

The radicals scavengers operate by interfering with the propagating step in the oxidative chain and this can be achieved by two routes:

(i) Reaction with propagating radicals (P^.^, PO^.^ POO^.^).
(ii) Reaction with resulting hydroperoxides which are the source of chain branching through the propagating process (Feldman 2002).

### Antioxidant

**Figure 36:**
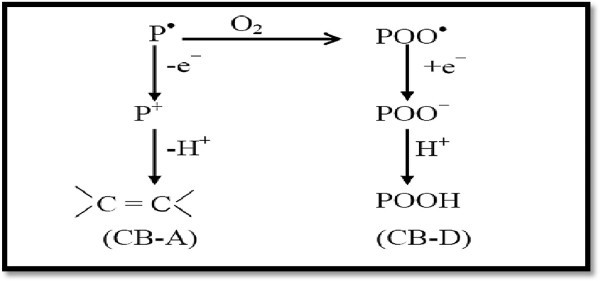
**Theory of antioxidant.**

The development of the auto-oxidation theory, in which the propagating radicals, alkyl, alkylperoxy (R^.^,ROO^.^) and the hydroperoxide (ROOH) are the key intermediates, has led to a comprehensive theory of antioxidant action. The two major anti-oxidant mechanisms are shown in Figure 36. The chain breaking donor (CB-D) and chain breaking acceptor (CB-A). Also an anti-oxidant can act by a preventive inhibition processes.

Many reducing agents e.g. hindered phenols and aromatic amines, which reduce the ROO^.^ to hydroperoxide in CB-D step have already been empirically selected and used for rubbers and by this time also for the newer plastics industry Cheremision (1997).

**Figure 37:**
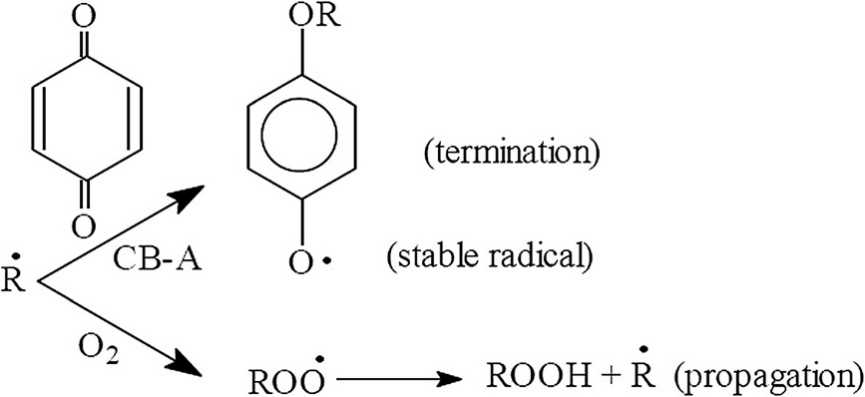
**Quinones retard oxidation process.**

Oxidizing agents, e.g. quinones which have been shown to be able to retard oxidation, can function as antioxidants (via a chain breaking acceptor process), if they can compete with oxygen for alkyl radicals Watson (1953), as shown in the following equation see Figure 37.

**Figure 38:**
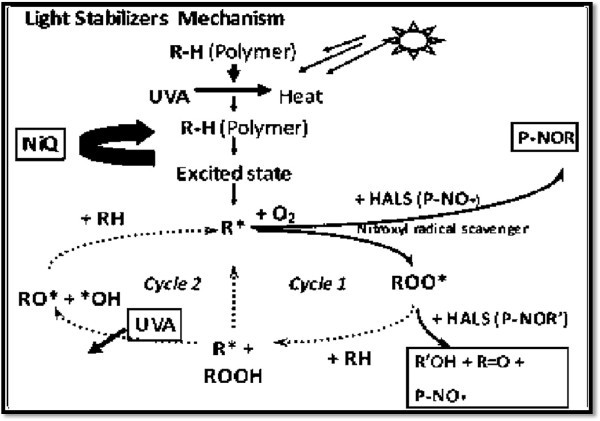
**Light stabilize function.**

Hydroperoxides (ROOH) are the primary molecular photooxidation products and the most dangerous chromophore in the photodegradation of polymers. Their deactivation by a hydroperoxide decomposing antioxidant (HDAO) or hindered amine stabilizer (HAS) reduces ROOH homolysis and consequently, chain initiation and transfer. The general light stabilizer function was shown in Figure 38.

### Photostabilization of polystyrene

The photostabilization process may be accomplished with commercial light stabilizers in addition to chalate complexes. The nature of the photostabilizer and polymer will determined the process and efficiency of photoprotection of the polymer film. The route by which photodegradation occurs and the importance of the impurities remaining after either polymerization or processing have to be known to determine the most satisfactory method of stabilization George (1974).

**Figure 39:**
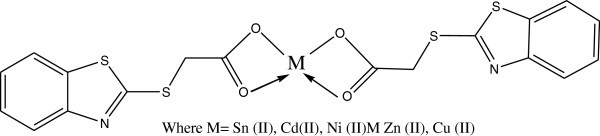
**Structure of 2-thioacitic acid benzothiazol complexes.**

**Figure 40:**
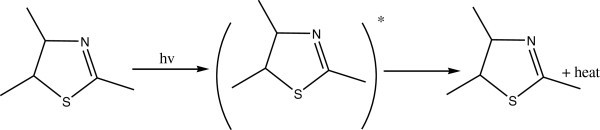
**Suggested process for photostabilization by benzothiazole as UV absorber, Yousif et al. (** 2012 **).**

**Figure 41:**
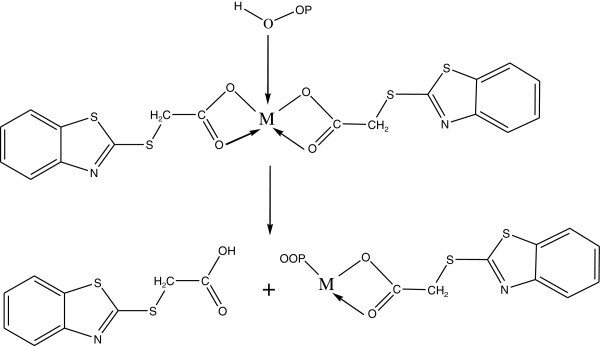
**Suggested process for photostabilization by benzothiazole complexes as peroxide decomposer, (Yousif et al.** 2012 **).**

**Figure 42:**
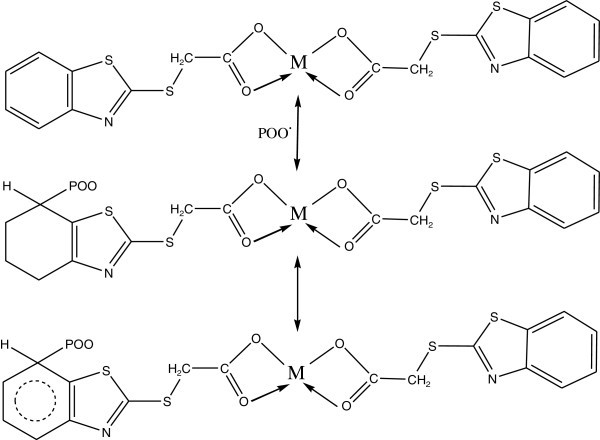
**Suggested mode for photostabilization by carboxylate complexes as radical scavengers.**

Complexes of 2-thioacitic acid benzothiazol have been used as additives to increase the photostabilization of polystyrene. The structures of these additives as shown in Figures 39, 40, 41, 42.

Two methods have been used to monitor the extent of photodegradation of polystyrene films. The first was by using FTIR spectra. The progress of photodegradation at different irradiation times was followed by observing the changes in carbonyl and hydroxyl peak intensities. Then, carbonyl (ICO) and hydroxyl (IOH) indices were calculated by comparison of the intensities of absorption peaks at 1720 and 3450 cm^-1^ with that of a reference peak at 1450 cm^-1^. This is called band index method. The second method involves measuring the average molecular weight using viscometry technique. These additives stabilize PS film through UV absorption or screening, and by peroxide decomposers, or radical scavengers Yousif et al. (2012).

**Figure 43:**
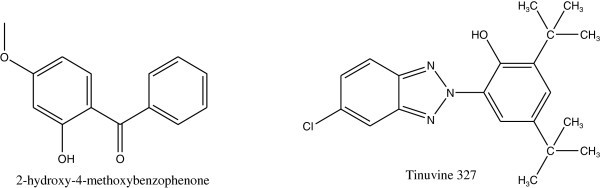
**Structures of 2-hydroxy-4-methoxybenzophenone and Tinuvin 327.**

The photochemical and photophysical protection for polystyrene by commercial UV absorbers such as 2-hydroxy-4-methoxybenzophenone and Tinuvin 327 involves triplet energy transfer from excited polymer carbonyl impurities groups in addition to UV screening George (1974). The structures of these compounds are shown in Figure 43.

**Figure 44:**
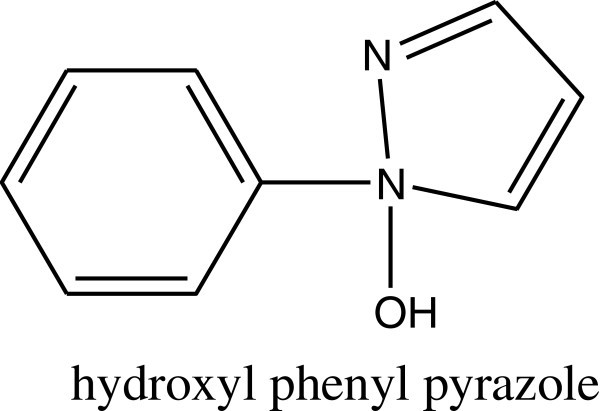
**Structure of hydroxyl phenyl pyrazole.**

Other categories of UV absorbers (hydroxyl phenyl pyrazole) have been shown to be able to act as photostabilizers to prevent oxidation or degradation of polystyrene. They may behave as an efficient triplet state quencher of carbonyl impurities in the polymer mainchain. Sastre et al. (1991) (see Figure 44).

**Figure 45:**
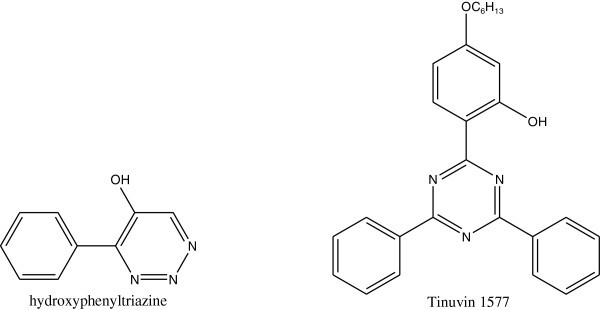
**Structures of Tinuvin 1577 and hydroxyphenyltriazine.**

Many commercial light stabilizers have been developed in an attempt to overcome the problem of photodegeradation of PS articles. The presence of various concentrations of Tinuvin 1577 Bottino et al. (1996), thiadiazole compounds Yousif et al. (2011a) and hydroxyphenyltriazine, additive effectively inhibits the surface photooxidation of PS Bottino et al. (1999). Also a new family of UV absorbers; the dihydroxyphenylpyrazoles, has shown to be effective as photostabilizers as they effectively prevent the photoxidative degradation of PS films Sastre et al.(2003). The compounds structures are shown in Figure 45.

**Figure 46:**
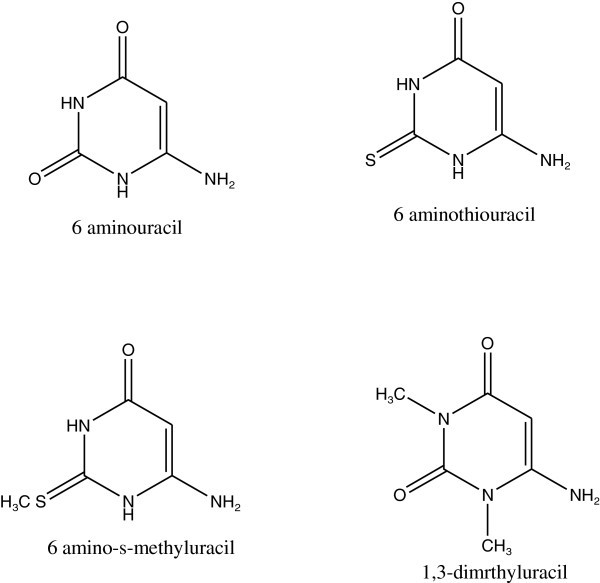
**Structures of uracil derivatives.**

Uracil derivatives have been used as photostabilizers for PS. The results reveal a higher stabilizing efficiency for the materials investigated relative to the two commercial UV -absorbers, phenyl salicylate and 2-hydroxybenzophenone derivative Rabie et al. (2008). The structures of uracil derivatives was shown in Figure 46.

**Figure 47:**
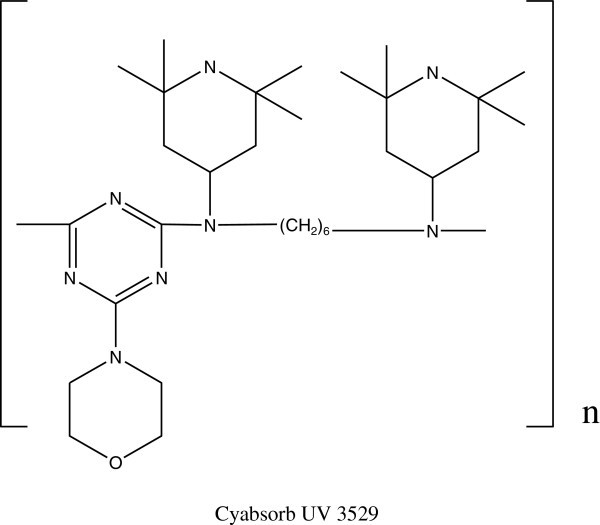
**Structure of Cyabsorb UV 3529.**

The photooxidation of PS decreased with addition of high molecular weight of HALS (linear peroxide amine type of new polymeric HALS) and the photostabilization efficiency was similar or better than that of Cyabsorb 3529 Sun et al. (2002) (Figure 47).

**Figure 48:**
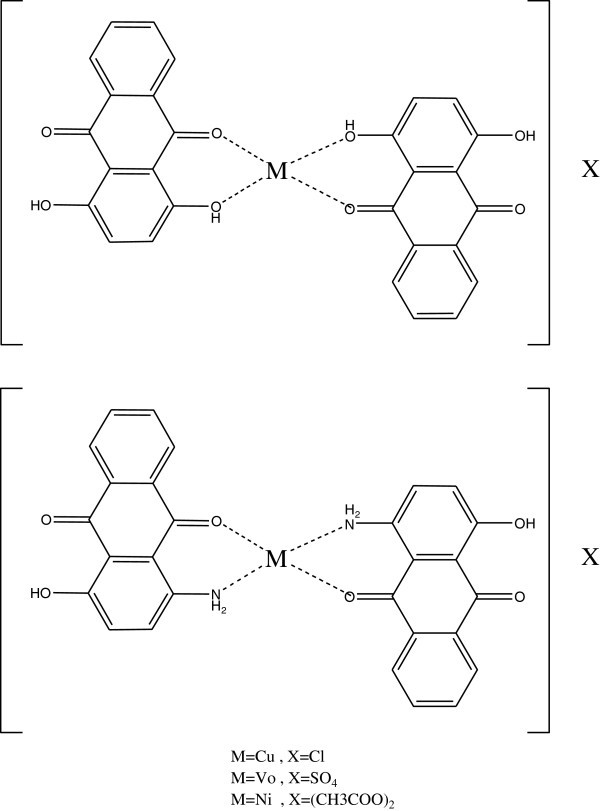
**Structure of anthraquinone derivatives.**

Photostabilization of PS films by anthraquinone derivatives and their complexes with copper(II), oxovanadium(IV) and nickel(II) ions has also been studied as shown in Figure 48 Abd et al. (2009).

Four methods have been used to monitor the degradation of polystyrene. There are: FTIR spectroscopy (measuring carbonyl and hydroxyl indexes), UV- visible spectroscopy, and solution viscosity (molecular weight). The last method for identification of degradation is by weight loss. The two anthraquinone derivatives and complexes provide good stabilization. Nickel and copper complexes provide better resistance to degradation than the uncomplexed anthraquinones Abd et al. (2009).

**Figure 49:**
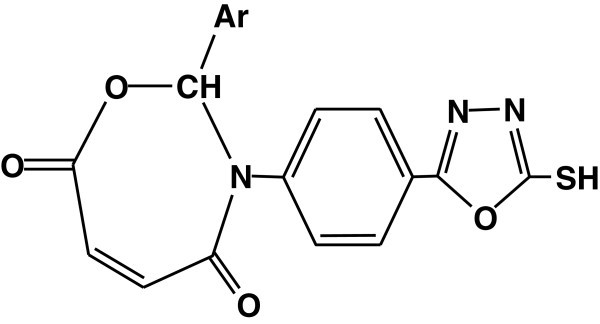
**Structure of 2,3dihydro-(5-mercapto-1,3,4-oxadiazol-2-yl)-phenyl-2-(substituted)-oxazepine- 4,7-dione.**

The photostabilizing effect of 2,3 dihydro-(5-mercapto-1,3,4-oxadiazol-2-yl)-phenyl-2-(substituted)-oxazepine- 4,7-dione compounds in PS has been reported. These additives stabilize PS films through UV absorption or screening, peroxide decomposition and radical scavenging Yousif et al. (2013) (see Figure 49).

The protection of polystyrene films by UV absorbers including Ni(II) chelate complexes has been studied. Acetylactonate of Ni(II), Cu(II), and Co (III) complexes are more efficient than the corresponding Fe(III) complex for the photostabilization of PS. George (1974); Aliwi *et al.* (2001).

## Conclusion

In recent years, the use of polymeric materials has rapidly increased but it is well established that rapid photodegradation of these materials is possible when they are exposed to natural weathering.

This review was talk about the photodegradation and photostabilization of polymers and especially in polystyrene. The “hydroperoxide” (POOH) is the most important initiator in the photooxidative process.

So most of the common polymers used in such applications contain photostabilizers to reduce photodamage and to ensure acceptable life times under outdoor exposure conditions. The photostabilization of polymers may be achieved in many ways. The following stabilizing systems have been developed, which depend on the action of stabilizer: light screeners, UV absorbers, excited-state quenchers, peroxide decomposers, and radical scavengers; of these, it is generally believed that excited-state quenchers, peroxide decomposers, and radical scavengers are the most effective.

However photoproduction will be enhanced if the additives can decompose –OOH, and possibly act as quenchers of some exited state in the early stages of the photodegradation

## Authors’ original submitted files for images

Below are the links to the authors’ original submitted files for images. Authors’ original file for figure 1 Authors’ original file for figure 2 Authors’ original file for figure 3 Authors’ original file for figure 4 Authors’ original file for figure 5 Authors’ original file for figure 6 Authors’ original file for figure 7 Authors’ original file for figure 8 Authors’ original file for figure 9 Authors’ original file for figure 10 Authors’ original file for figure 11 Authors’ original file for figure 12 Authors’ original file for figure 13 Authors’ original file for figure 14 Authors’ original file for figure 15 Authors’ original file for figure 16 Authors’ original file for figure 17 Authors’ original file for figure 18 Authors’ original file for figure 19 Authors’ original file for figure 20 Authors’ original file for figure 21 Authors’ original file for figure 22 Authors’ original file for figure 23 Authors’ original file for figure 24 Authors’ original file for figure 25 Authors’ original file for figure 26 Authors’ original file for figure 27 Authors’ original file for figure 28 Authors’ original file for figure 29 Authors’ original file for figure 30 Authors’ original file for figure 31 Authors’ original file for figure 32 Authors’ original file for figure 33 Authors’ original file for figure 34 Authors’ original file for figure 35 Authors’ original file for figure 36 Authors’ original file for figure 37 Authors’ original file for figure 38 Authors’ original file for figure 39 Authors’ original file for figure 40 Authors’ original file for figure 41 Authors’ original file for figure 42 Authors’ original file for figure 43 Authors’ original file for figure 44 Authors’ original file for figure 45 Authors’ original file for figure 46 Authors’ original file for figure 47 Authors’ original file for figure 48 Authors’ original file for figure 49 Authors’ original file for figure 50 Authors’ original file for figure 51
