# Supplementary material for: Photodegradation and photostabilization of polymers, especially polystyrene: review
Source: Springerplus. 2013 Aug 23;2:398. doi: 10.1186/2193-1801-2-398 (PMC4320144; doi:10.1186/2193-1801-2-398)
Supplement: Supplementary file 1 — Authors’ original file for figure 1 [file 40064_2013_1415_MOESM1_ESM.pdf]

## (i)Chain initiation:

Hydroperoxide (POOH)

Carbonyl compounds (c=o)

Catalyst residue ($Ti^{n}$ , $V^{+n}$ etc)

Charge transfer complexes (PH , $O_2$)

$$\left.\begin{array}{l}\text{Hydroperoxide (POOH)}\\ \text{Carbonyl compounds (c=o)}\\ \text{Catalyst residue } (Ti^{n}, V^{+n} \text{ etc})\\ \text{Charge transfer complexes } (PH, O_2)\end{array}\right\} \xrightarrow[M^{+2},\ M^{+3}]{\text{heat , hv}} P^{\cdot}, POO^{\cdot}, OH^{\cdot}, HO_2^{\cdot} \quad \text{radical}$$

## (ii)Chain propagation:

$$P^{\cdot} + O_2 \xrightarrow[M^{+2},\ M^{+3}]{\text{heat , hv}} POO^{\cdot}$$

$$POO^{\cdot} + PH \xrightarrow[M^{+2},\ M^{+3}]{\text{heat , hv}} POOH + P^{\cdot}$$

$$POOH \longrightarrow PO^{\cdot} + {}^{\cdot}OH$$

$$2POOH \longrightarrow POO^{\cdot} + PO +$$

$$PO^{\cdot} + PH \longrightarrow POH + P^{\cdot}$$

$$HO^{\cdot} + PH \longrightarrow H_2O + P^{\cdot}$$

## (iii)Chain termination:

$$\left.\begin{array}{l} P^{\cdot} + P^{\cdot}\\ P^{\cdot} + POO^{\cdot}\\ POO^{\cdot} + POO^{\cdot}\end{array}\right\} \longrightarrow \text{non radical products}$$

e.t.c

**Where $P^{\cdot}$ is polymer radical , $M^{+2}$ is metal ion , and PH is polymer molecule**
