# Supplementary figures and images for: Photodegradation and photostabilization of polymers, especially polystyrene: review
Source: Springerplus. 2013 Aug 23;2:398. doi: 10.1186/2193-1801-2-398 (PMC4320144; doi:10.1186/2193-1801-2-398)

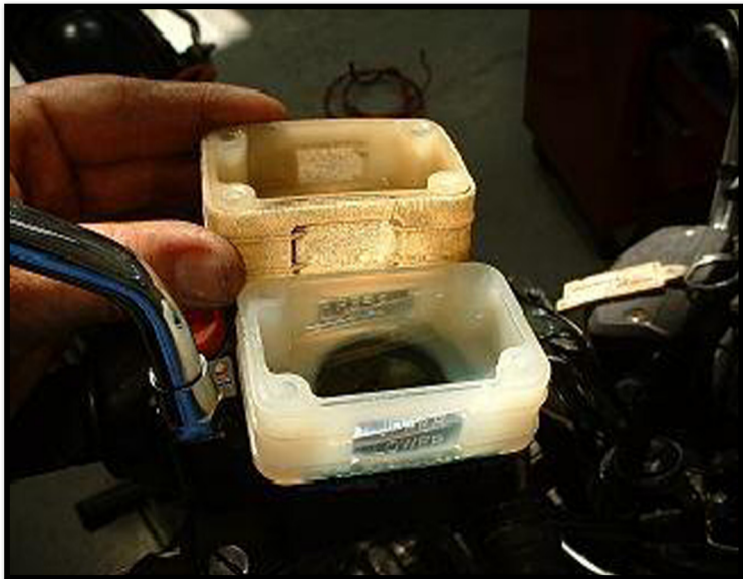

Supplement: Supplementary file 2 — Authors’ original file for figure 2 [file 40064_2013_1415_MOESM2_ESM.pdf]

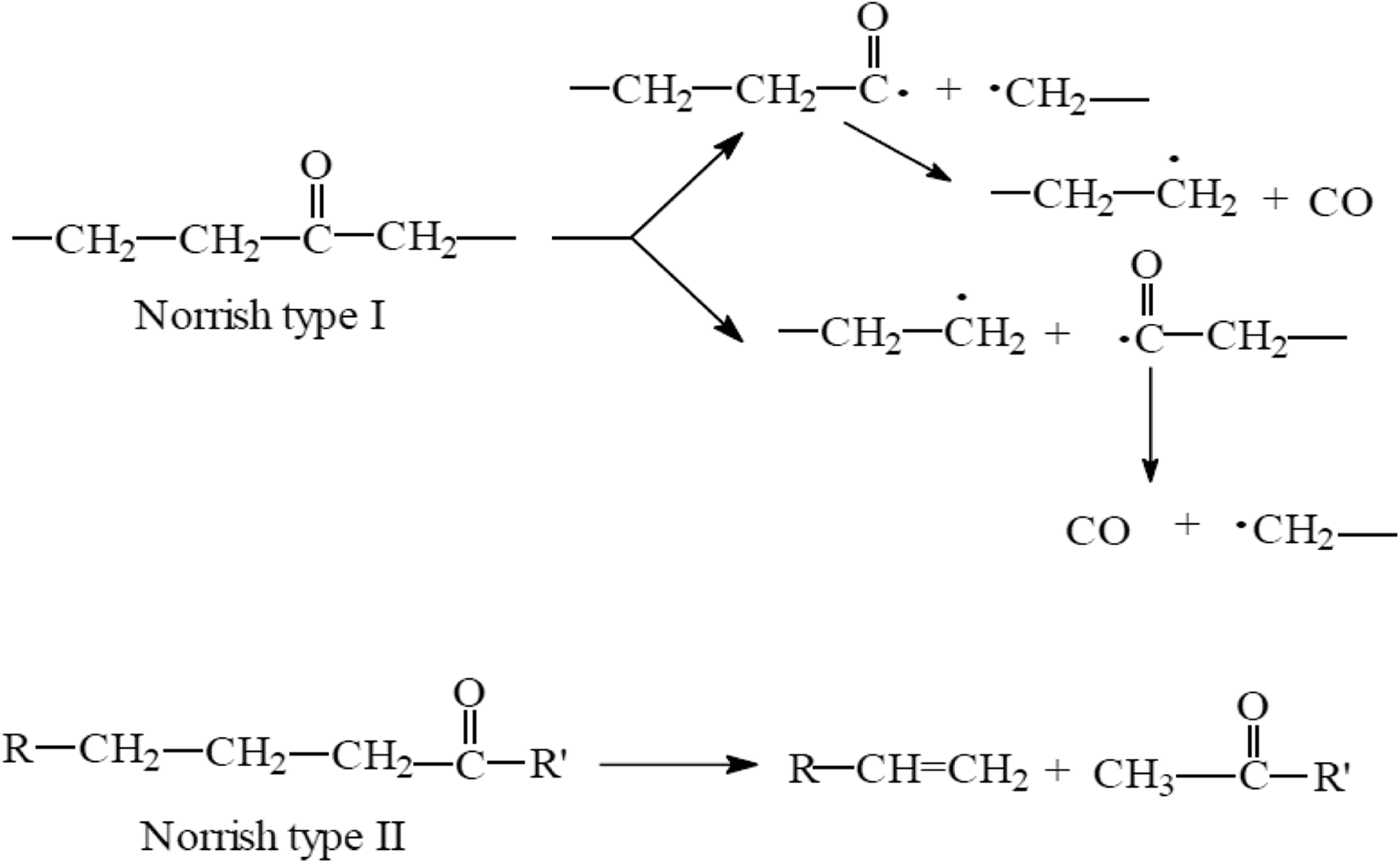

Supplement: Supplementary file 3 — Authors’ original file for figure 3 [file 40064_2013_1415_MOESM3_ESM.tiff]

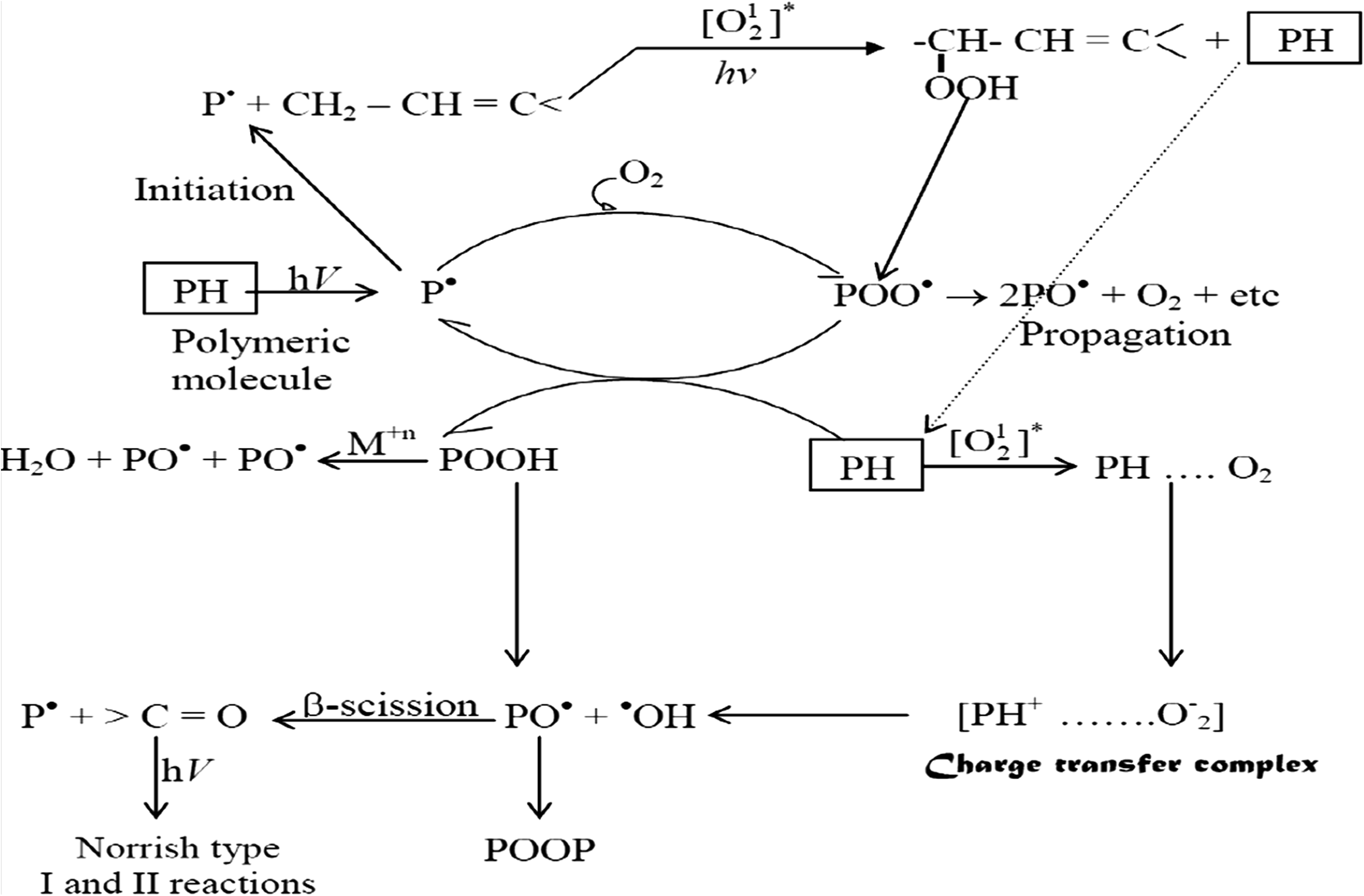

Supplement: Supplementary file 4 — Authors’ original file for figure 4 [file 40064_2013_1415_MOESM4_ESM.tiff]

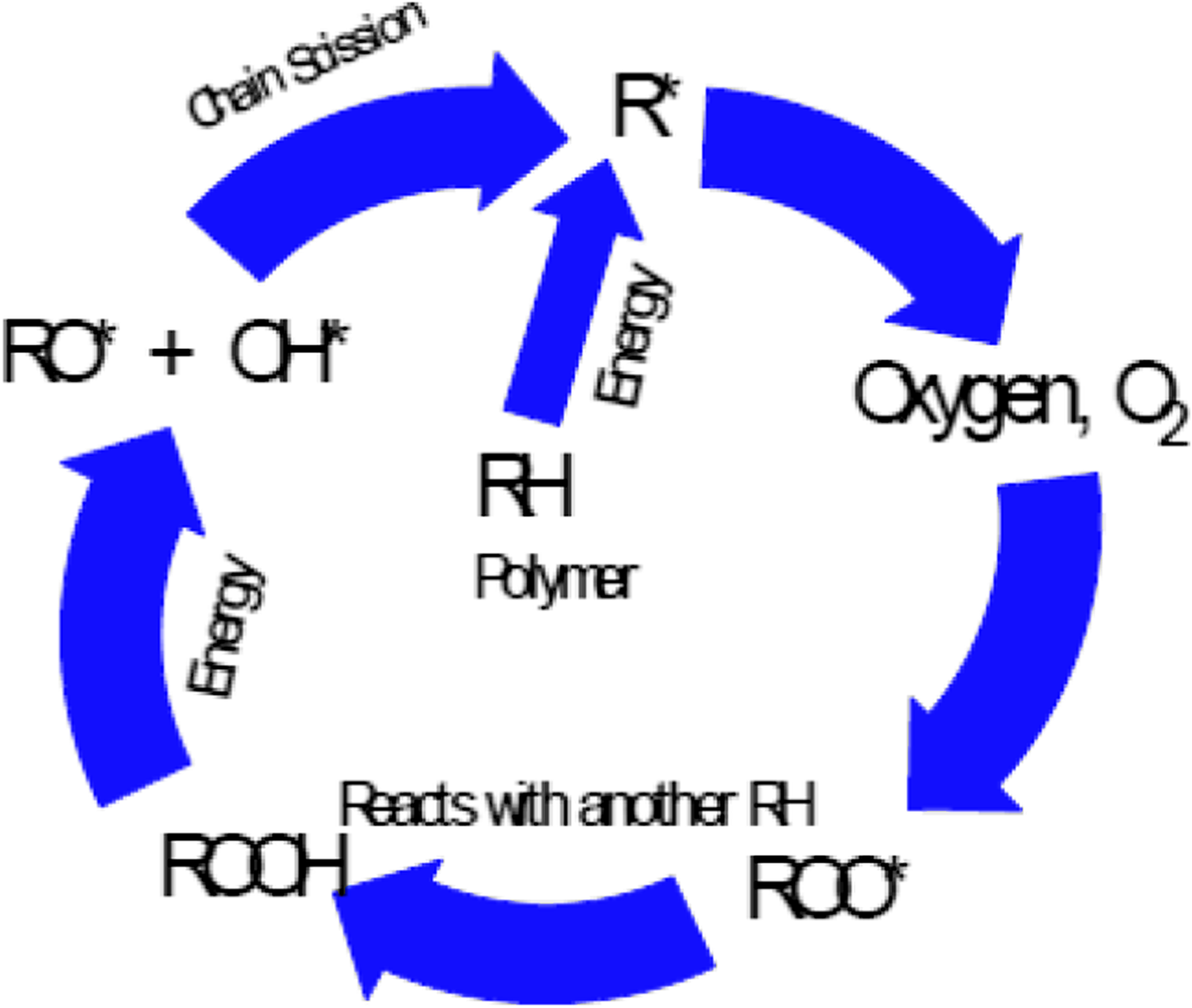

Supplement: Supplementary file 5 — Authors’ original file for figure 5 [file 40064_2013_1415_MOESM5_ESM.tiff]

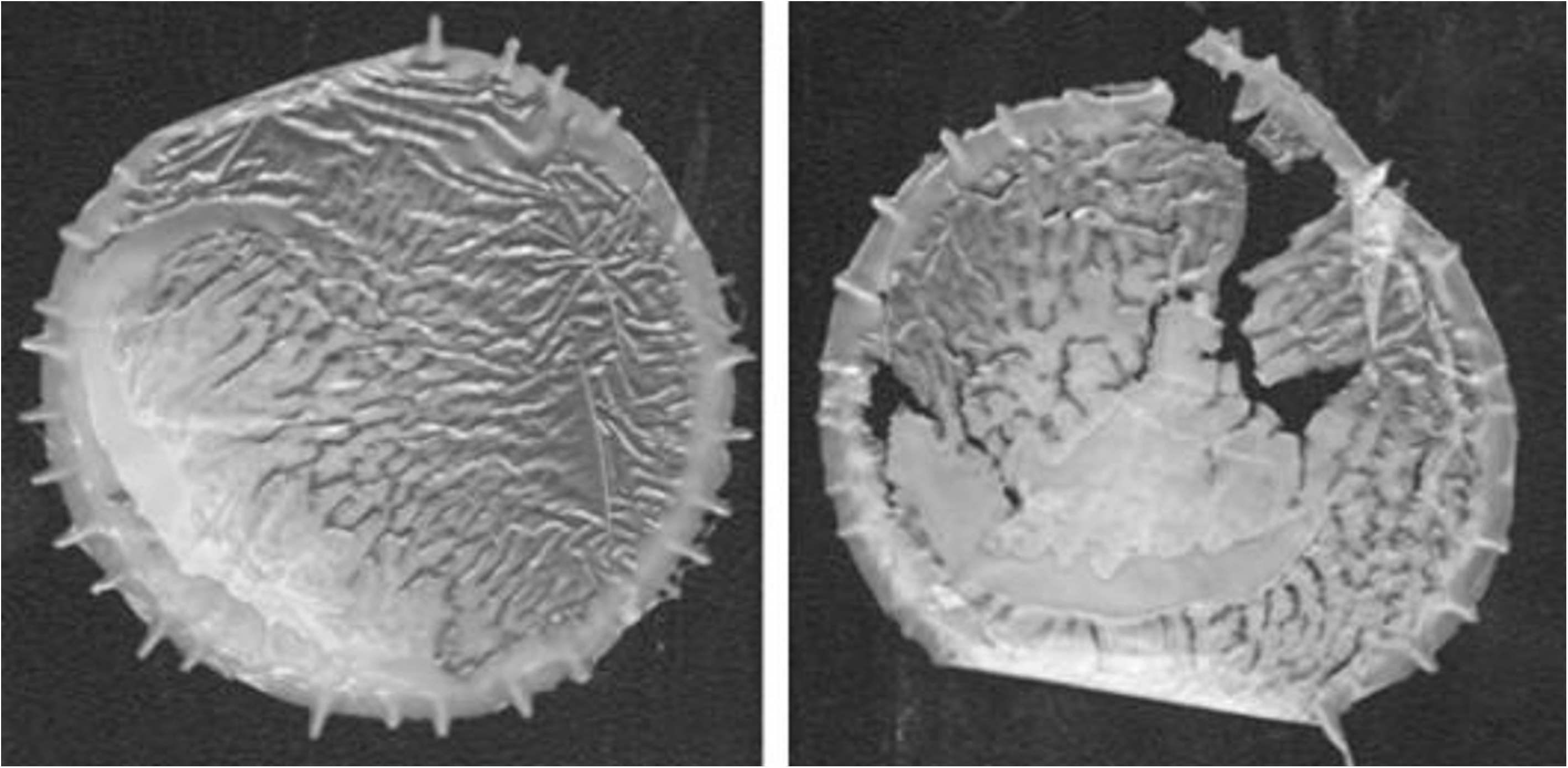

Supplement: Supplementary file 6 — Authors’ original file for figure 6 [file 40064_2013_1415_MOESM6_ESM.tiff]

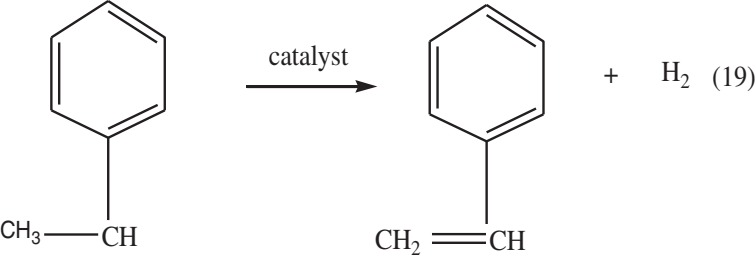

Supplement: Supplementary file 7 — Authors’ original file for figure 7 [file 40064_2013_1415_MOESM7_ESM.pdf]

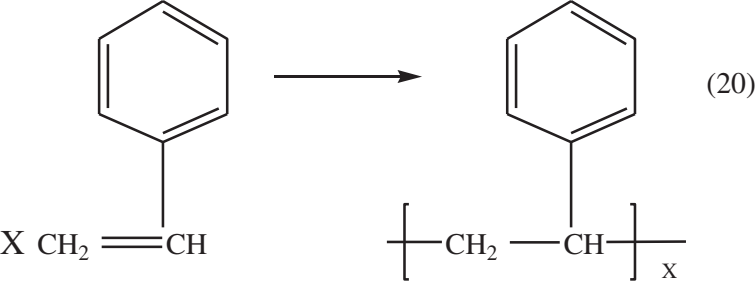

Supplement: Supplementary file 8 — Authors’ original file for figure 8 [file 40064_2013_1415_MOESM8_ESM.pdf]

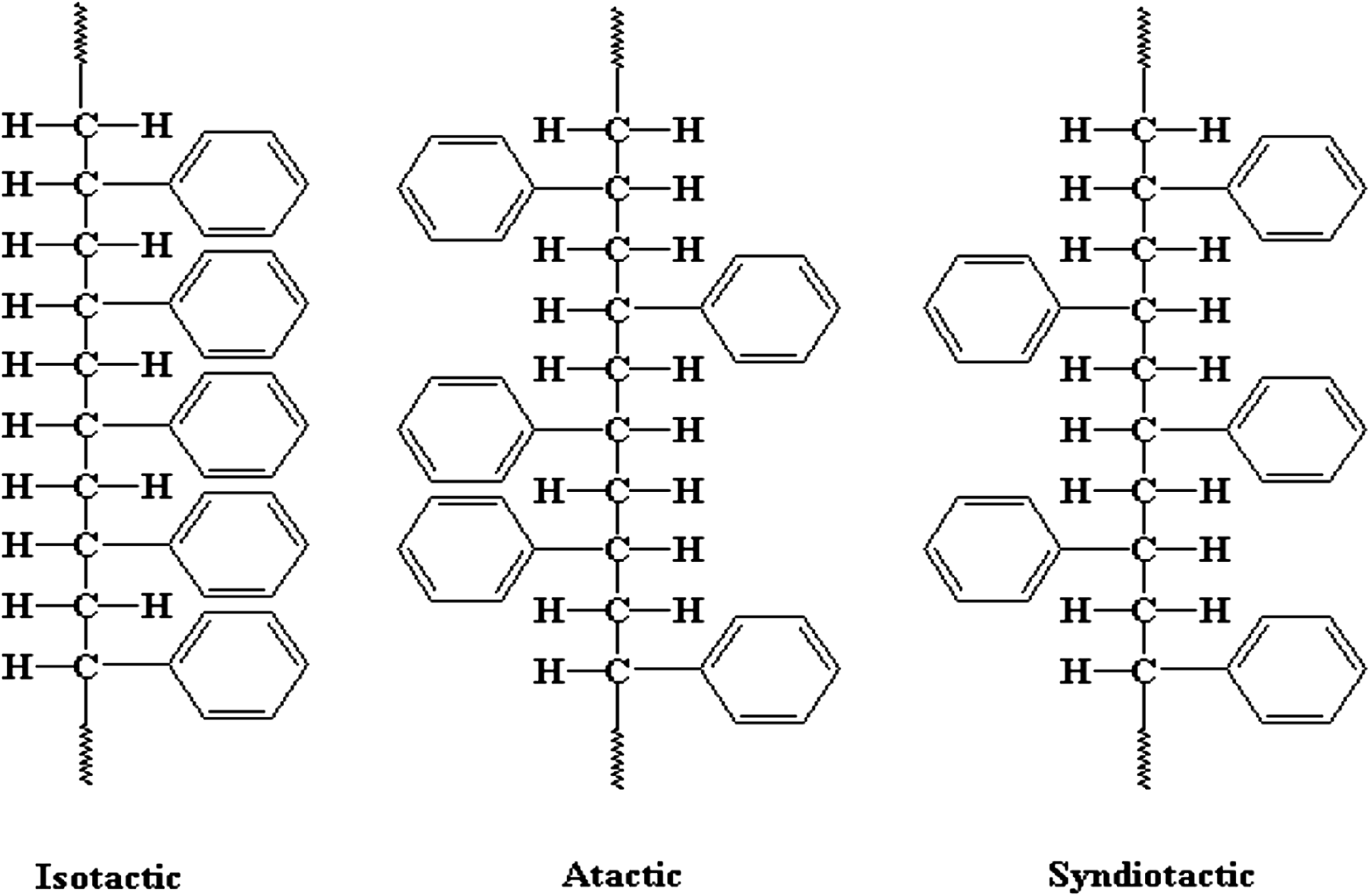

Supplement: Supplementary file 9 — Authors’ original file for figure 9 [file 40064_2013_1415_MOESM9_ESM.tiff]

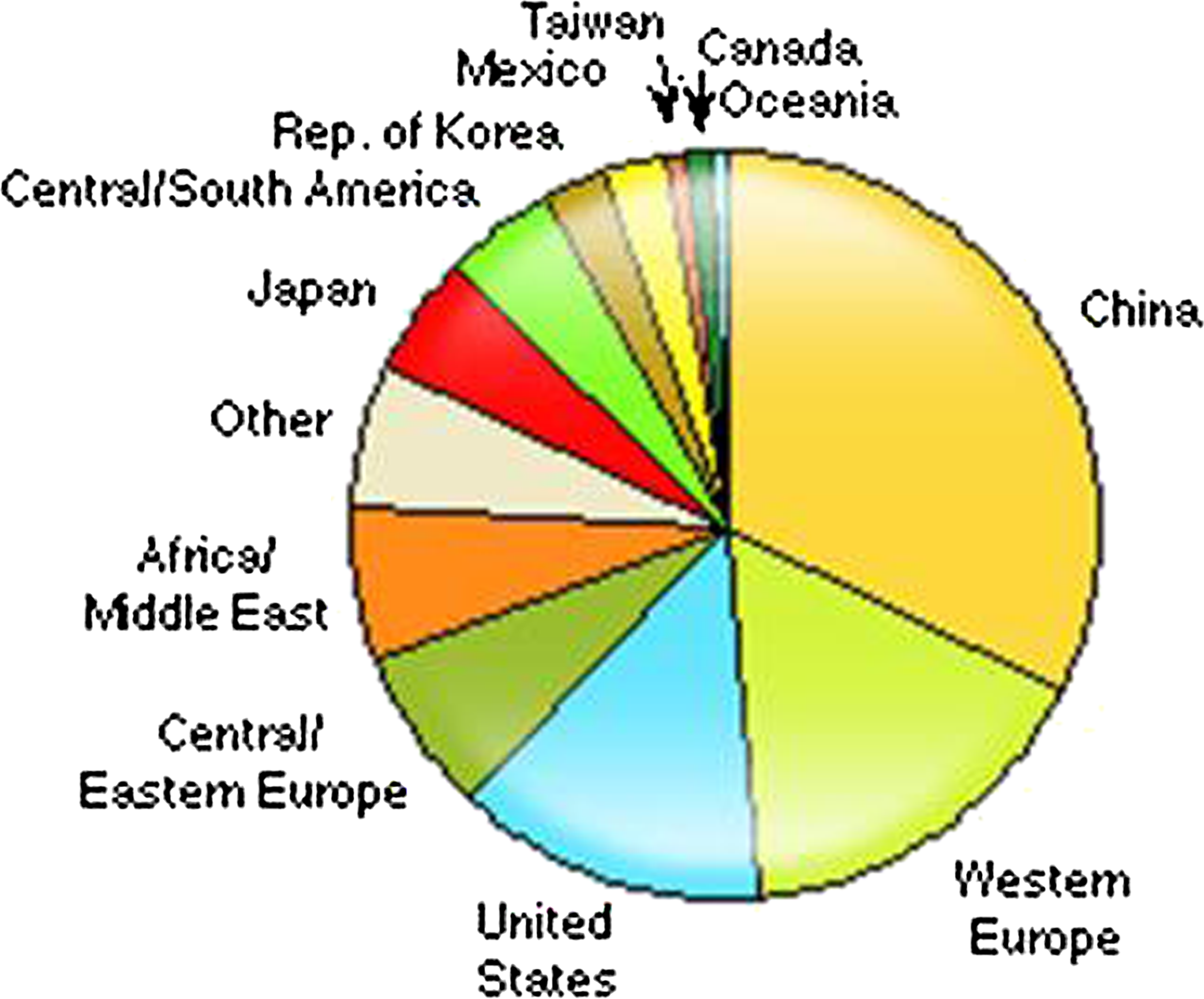

Supplement: Supplementary file 10 — Authors’ original file for figure 10 [file 40064_2013_1415_MOESM10_ESM.tiff]

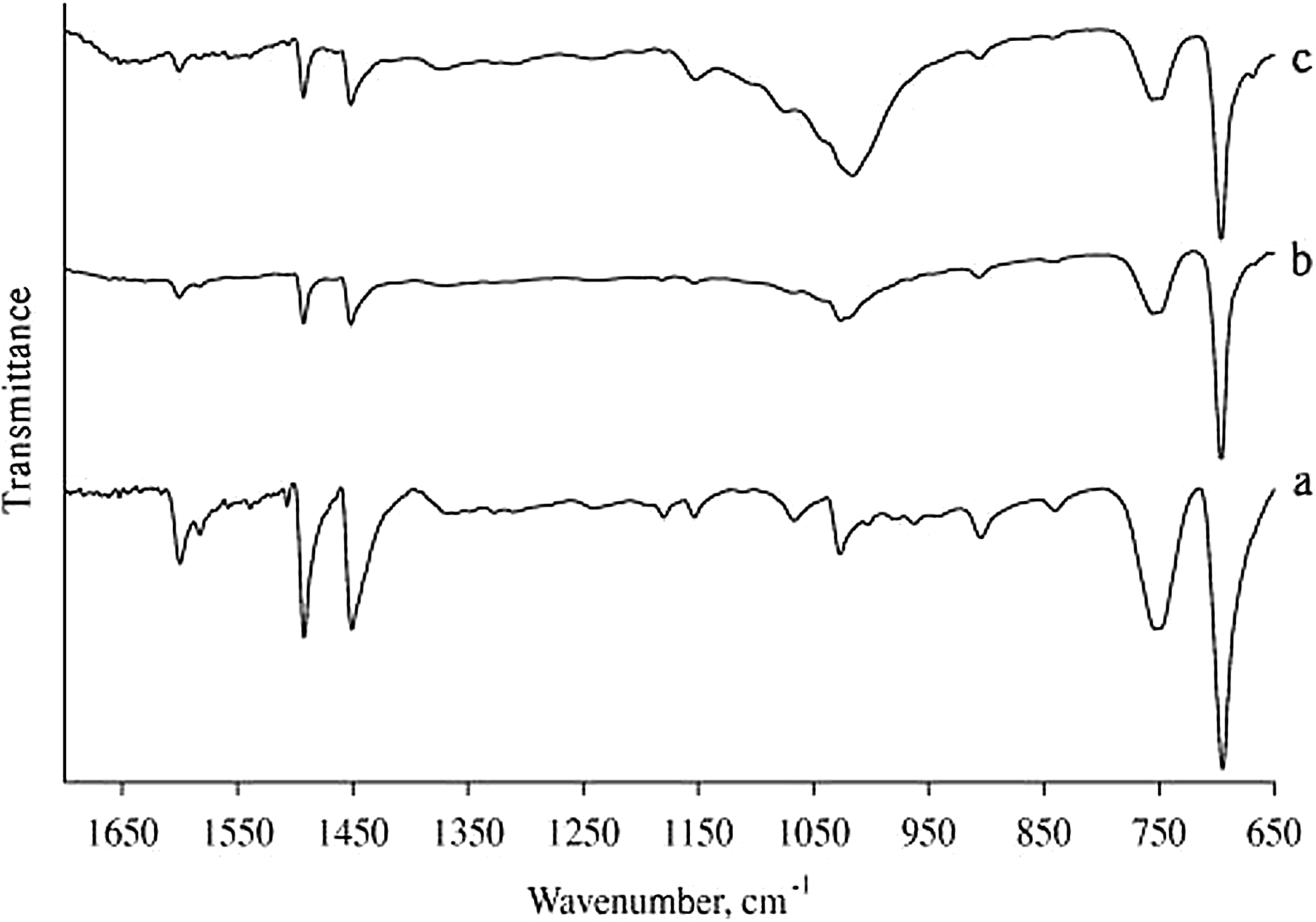

Supplement: Supplementary file 11 — Authors’ original file for figure 11 [file 40064_2013_1415_MOESM11_ESM.tiff]

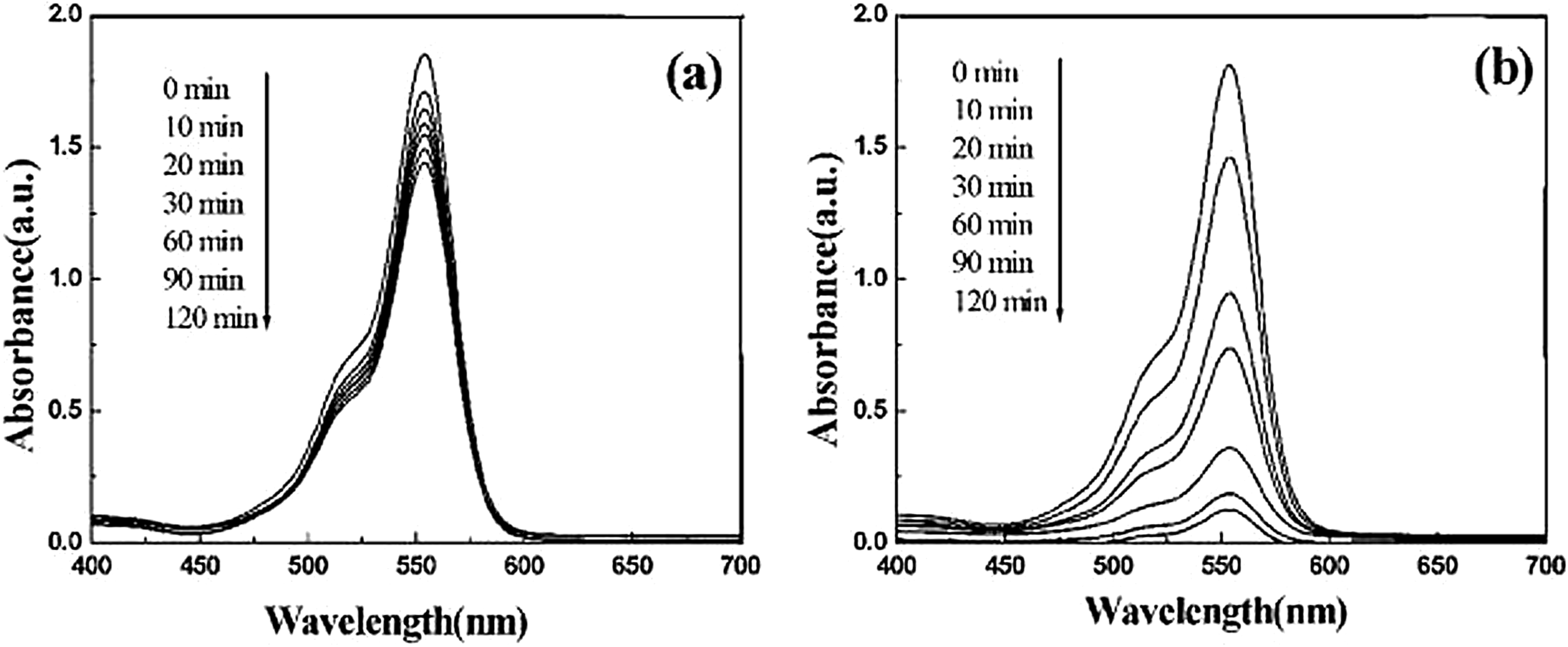

Supplement: Supplementary file 12 — Authors’ original file for figure 12 [file 40064_2013_1415_MOESM12_ESM.tiff]

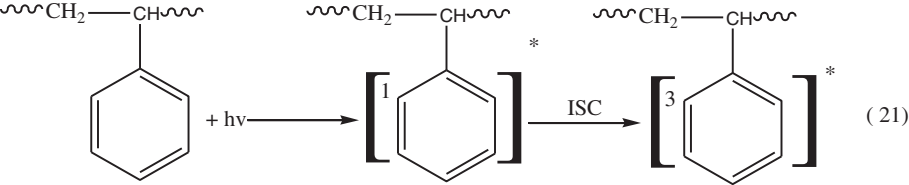

Supplement: Supplementary file 13 — Authors’ original file for figure 13 [file 40064_2013_1415_MOESM13_ESM.pdf]

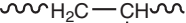

3

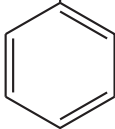

\*

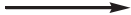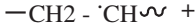

+

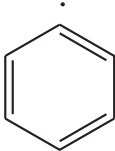

( 22 )

Supplement: Supplementary file 14 — Authors’ original file for figure 14 [file 40064_2013_1415_MOESM14_ESM.pdf]

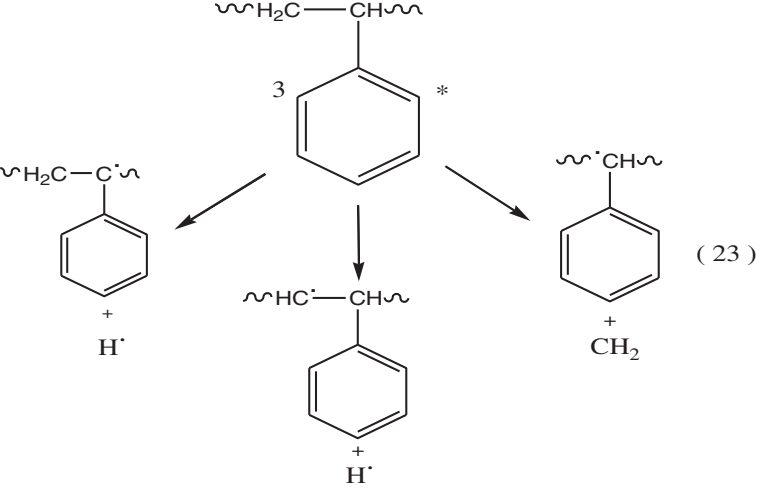

Supplement: Supplementary file 15 — Authors’ original file for figure 15 [file 40064_2013_1415_MOESM15_ESM.pdf]

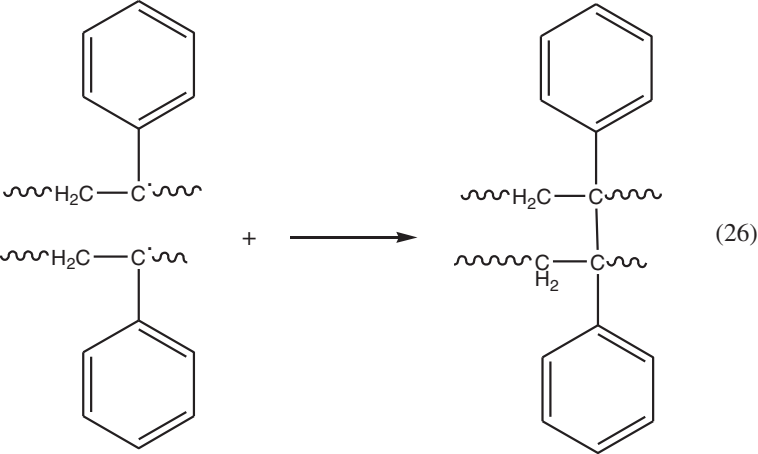

Supplement: Supplementary file 16 — Authors’ original file for figure 16 [file 40064_2013_1415_MOESM16_ESM.pdf]

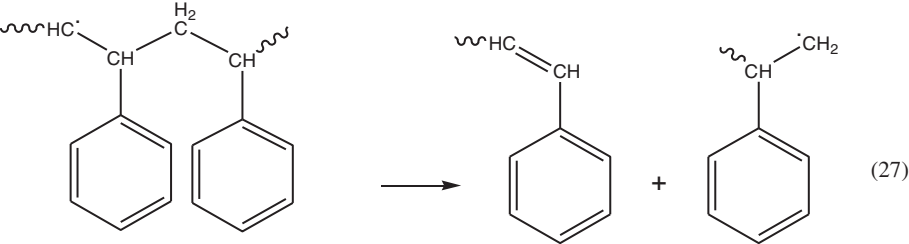

Supplement: Supplementary file 17 — Authors’ original file for figure 17 [file 40064_2013_1415_MOESM17_ESM.pdf]

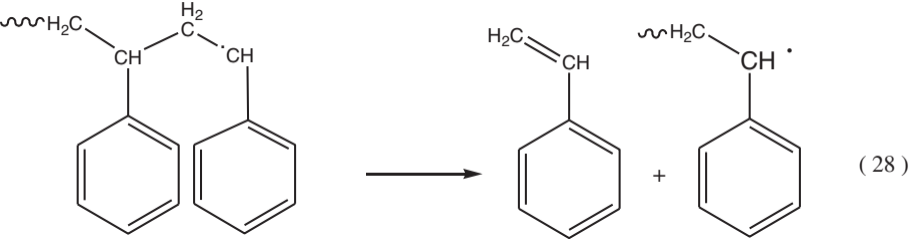

Supplement: Supplementary file 18 — Authors’ original file for figure 18 [file 40064_2013_1415_MOESM18_ESM.pdf]

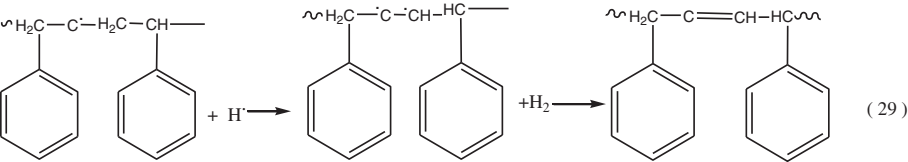

Supplement: Supplementary file 19 — Authors’ original file for figure 19 [file 40064_2013_1415_MOESM19_ESM.pdf]

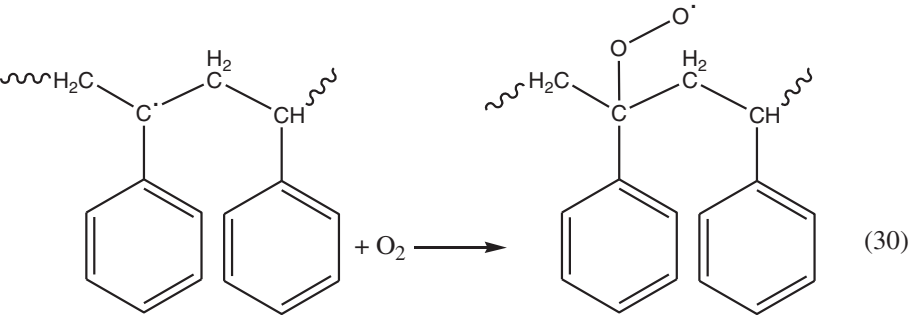

Supplement: Supplementary file 20 — Authors’ original file for figure 20 [file 40064_2013_1415_MOESM20_ESM.pdf]

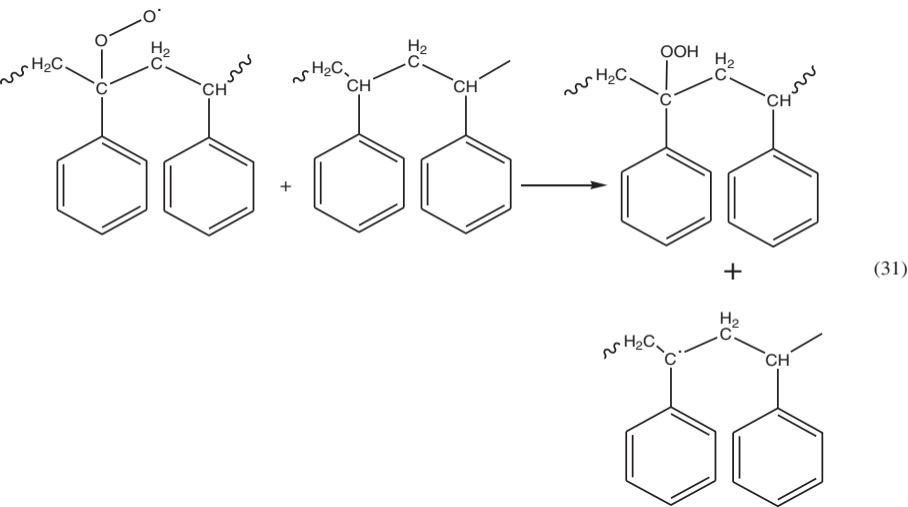

Supplement: Supplementary file 21 — Authors’ original file for figure 21 [file 40064_2013_1415_MOESM21_ESM.pdf]

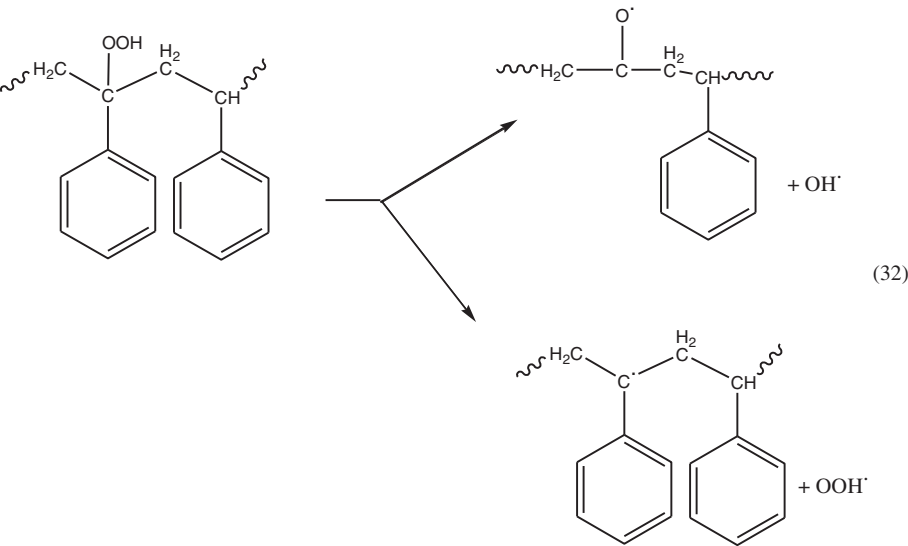

Supplement: Supplementary file 22 — Authors’ original file for figure 22 [file 40064_2013_1415_MOESM22_ESM.pdf]

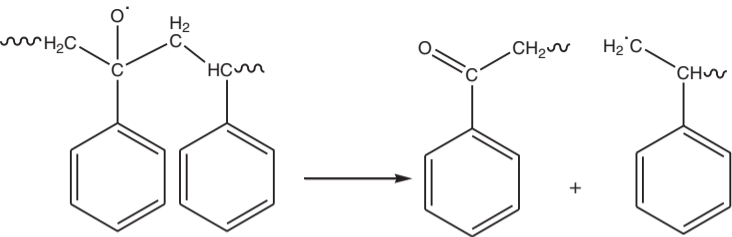

(33)

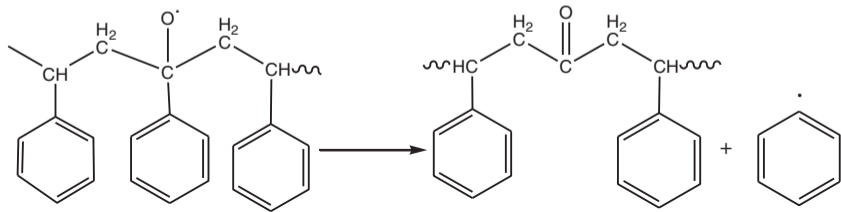

Supplement: Supplementary file 23 — Authors’ original file for figure 23 [file 40064_2013_1415_MOESM23_ESM.pdf]

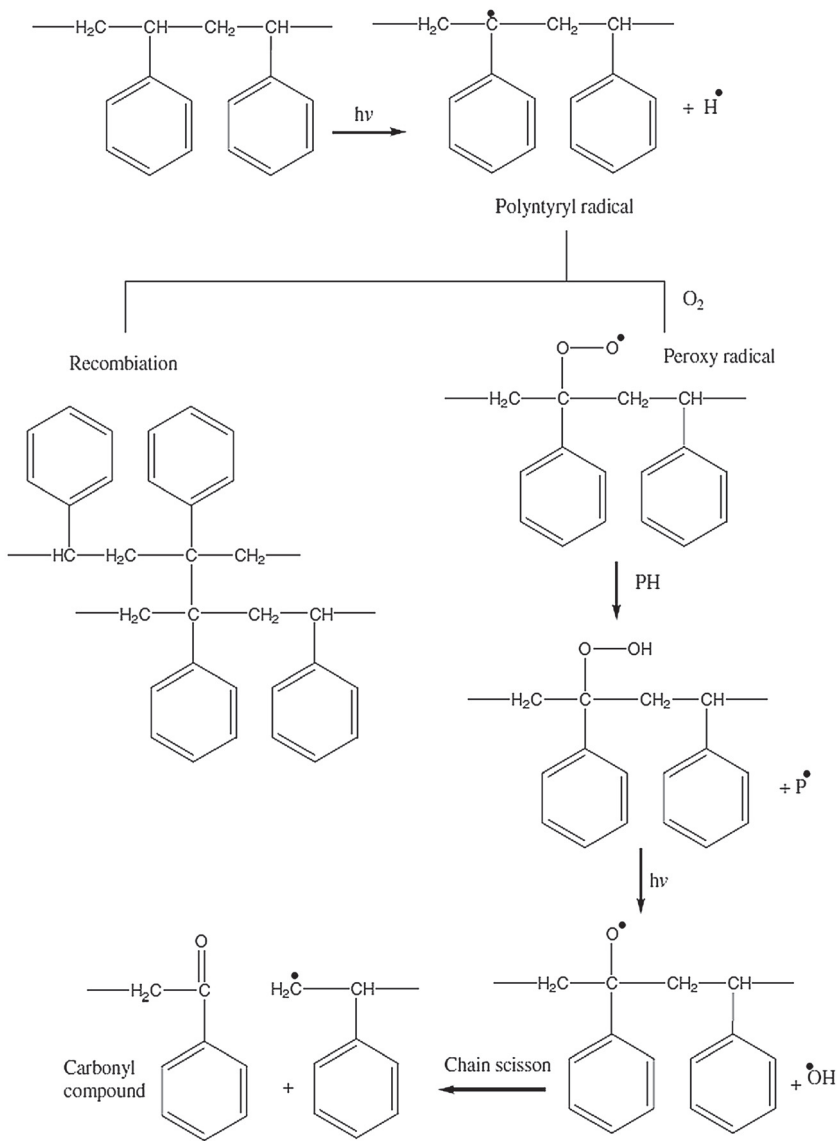

Supplement: Supplementary file 24 — Authors’ original file for figure 24 [file 40064_2013_1415_MOESM24_ESM.pdf]

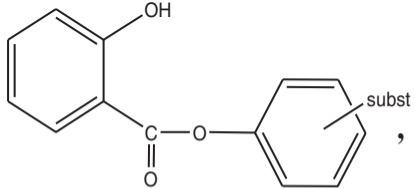

1

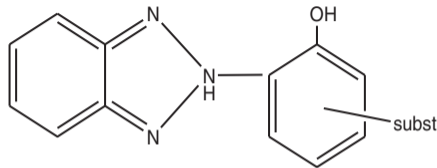

2

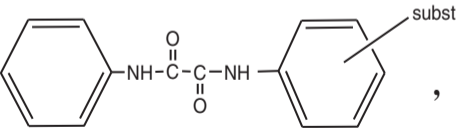

3

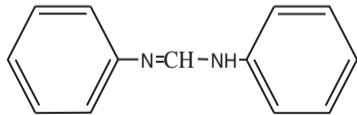

4

Supplement: Supplementary file 25 — Authors’ original file for figure 25 [file 40064_2013_1415_MOESM25_ESM.pdf]

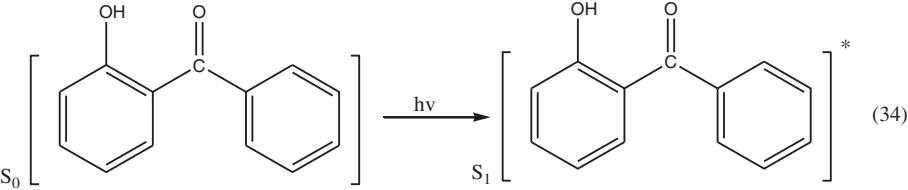

Supplement: Supplementary file 26 — Authors’ original file for figure 26 [file 40064_2013_1415_MOESM26_ESM.pdf]

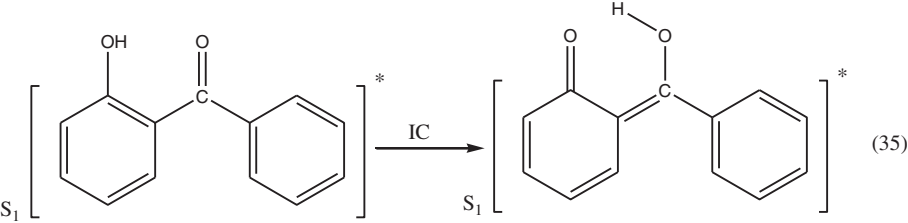

Supplement: Supplementary file 27 — Authors’ original file for figure 27 [file 40064_2013_1415_MOESM27_ESM.pdf]

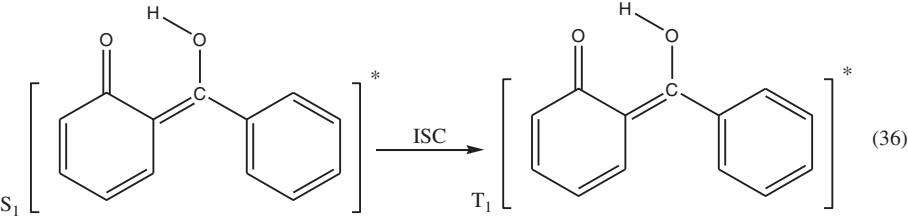

Supplement: Supplementary file 28 — Authors’ original file for figure 28 [file 40064_2013_1415_MOESM28_ESM.pdf]

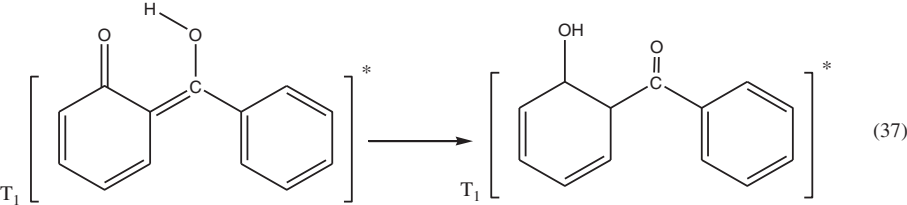

Supplement: Supplementary file 29 — Authors’ original file for figure 29 [file 40064_2013_1415_MOESM29_ESM.pdf]

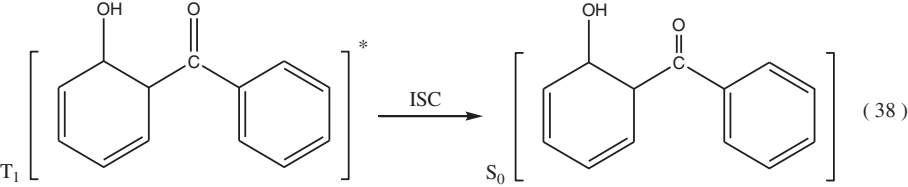

Supplement: Supplementary file 30 — Authors’ original file for figure 30 [file 40064_2013_1415_MOESM30_ESM.pdf]

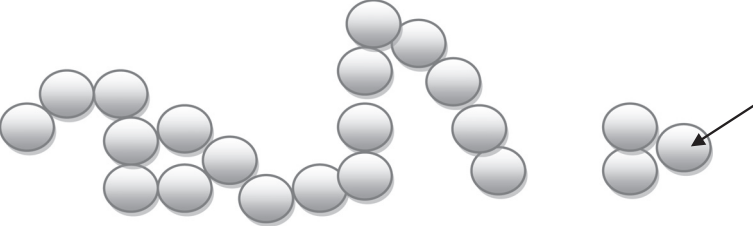

Supplement: Supplementary file 31 — Authors’ original file for figure 31 [file 40064_2013_1415_MOESM31_ESM.pdf]

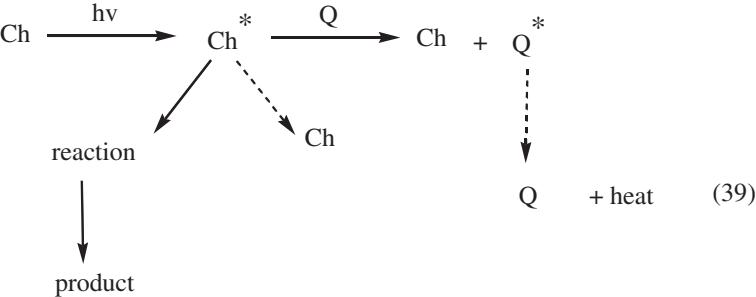

Supplement: Supplementary file 32 — Authors’ original file for figure 32 [file 40064_2013_1415_MOESM32_ESM.pdf]

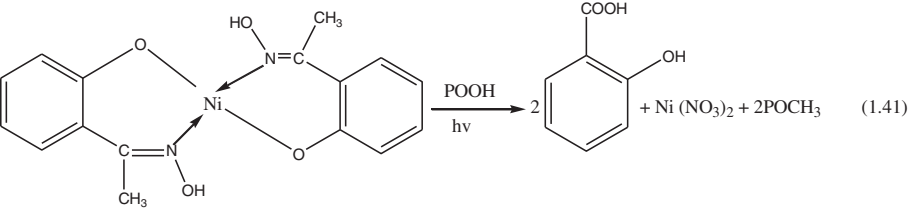

Supplement: Supplementary file 33 — Authors’ original file for figure 33 [file 40064_2013_1415_MOESM33_ESM.pdf]

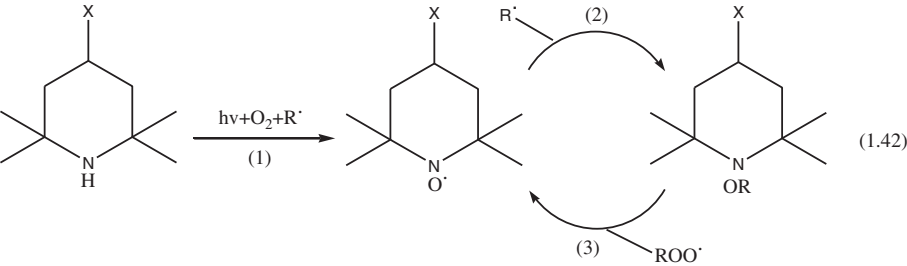

Supplement: Supplementary file 34 — Authors’ original file for figure 34 [file 40064_2013_1415_MOESM34_ESM.pdf]

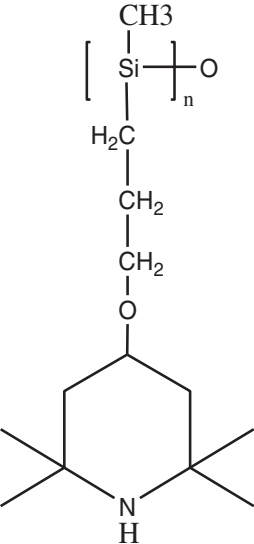

Uvasil992

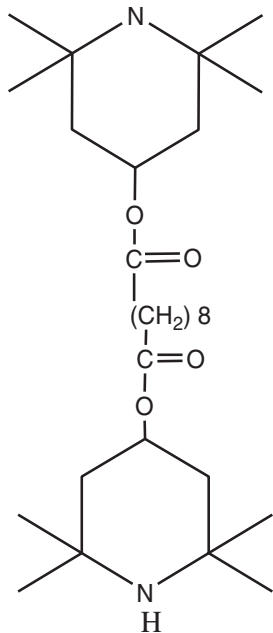

Tinouvin770

Supplement: Supplementary file 35 — Authors’ original file for figure 35 [file 40064_2013_1415_MOESM35_ESM.pdf]

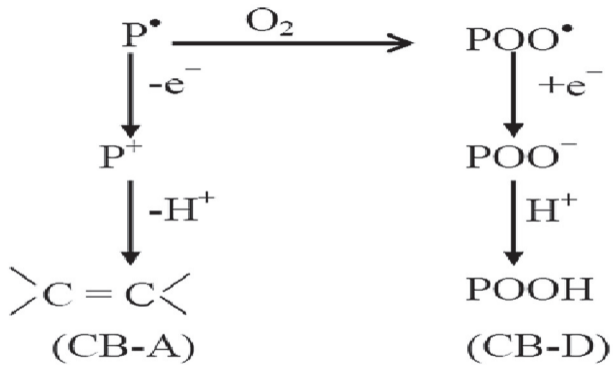

Supplement: Supplementary file 36 — Authors’ original file for figure 36 [file 40064_2013_1415_MOESM36_ESM.pdf]

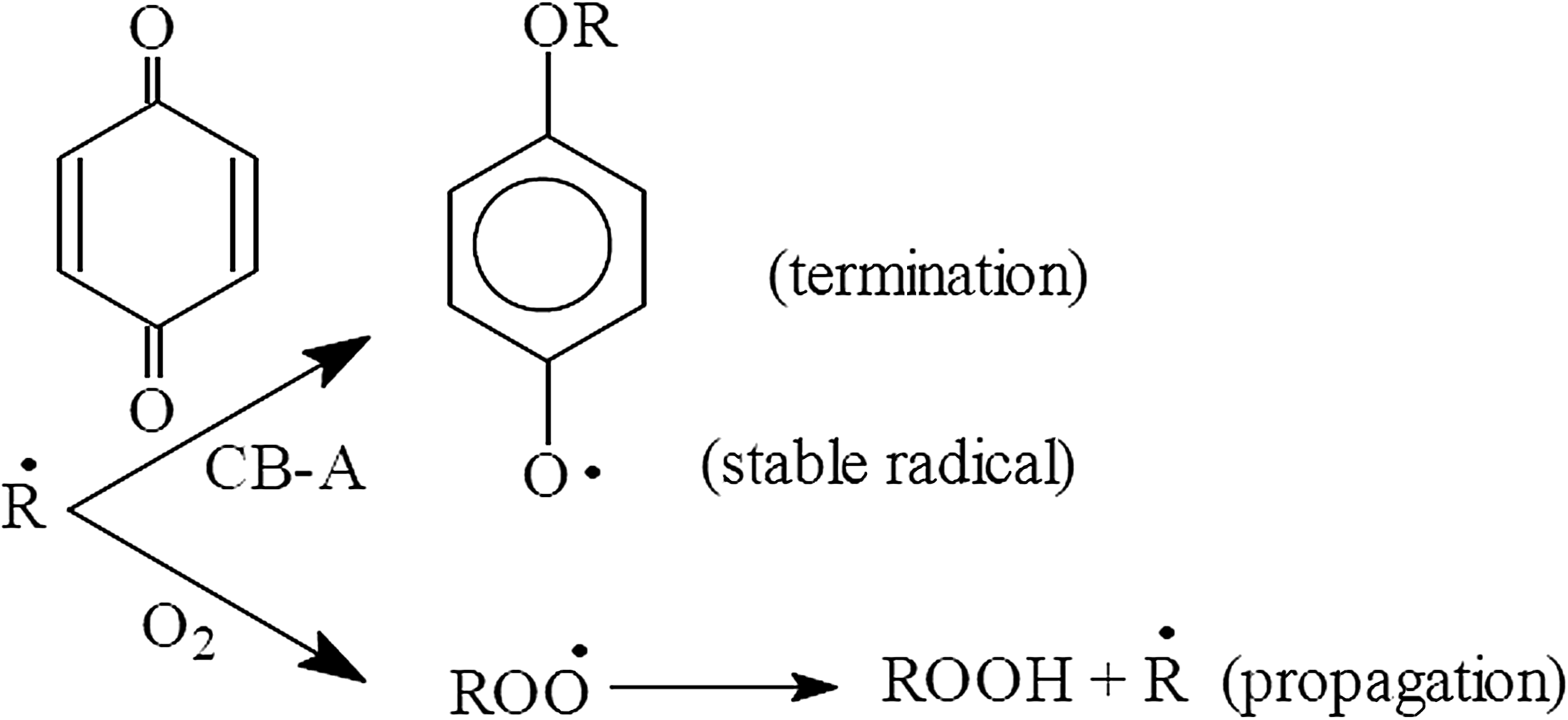

Supplement: Supplementary file 37 — Authors’ original file for figure 37 [file 40064_2013_1415_MOESM37_ESM.tiff]

# Light Stabilizers Mechanism

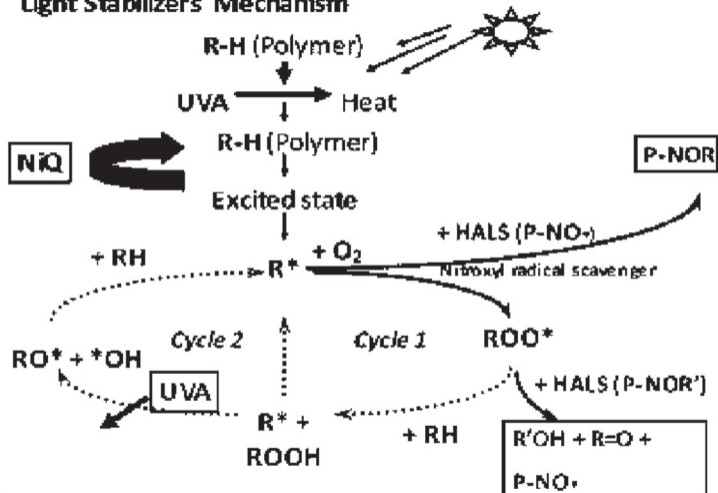

Supplement: Supplementary file 38 — Authors’ original file for figure 38 [file 40064_2013_1415_MOESM38_ESM.pdf]

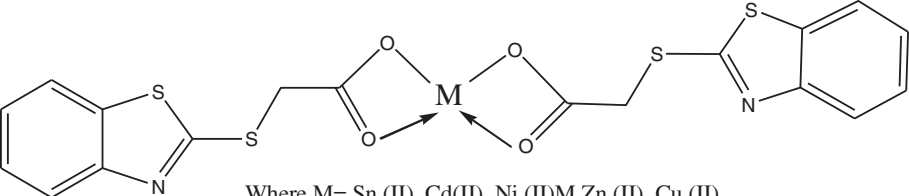

Supplement: Supplementary file 39 — Authors’ original file for figure 39 [file 40064_2013_1415_MOESM39_ESM.pdf]

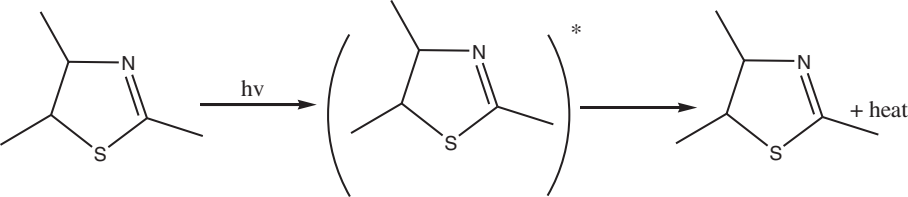

Supplement: Supplementary file 40 — Authors’ original file for figure 40 [file 40064_2013_1415_MOESM40_ESM.pdf]

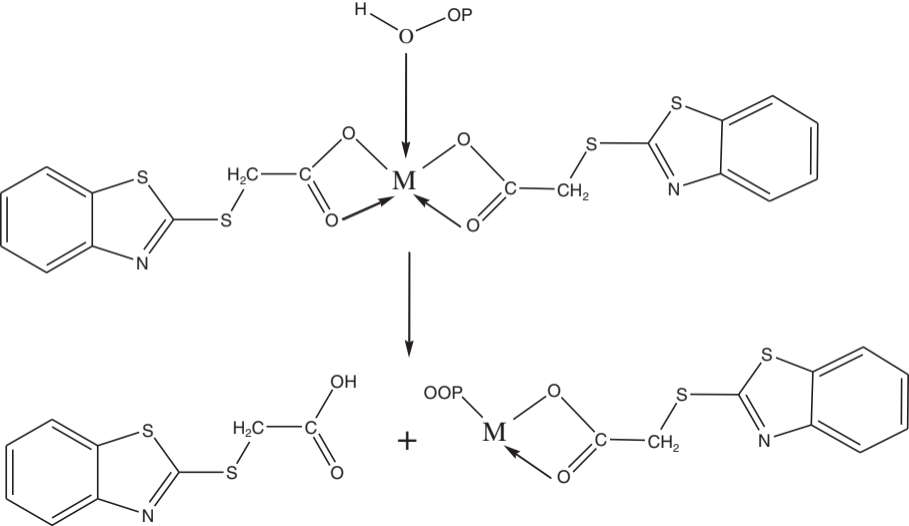

Supplement: Supplementary file 41 — Authors’ original file for figure 41 [file 40064_2013_1415_MOESM41_ESM.pdf]

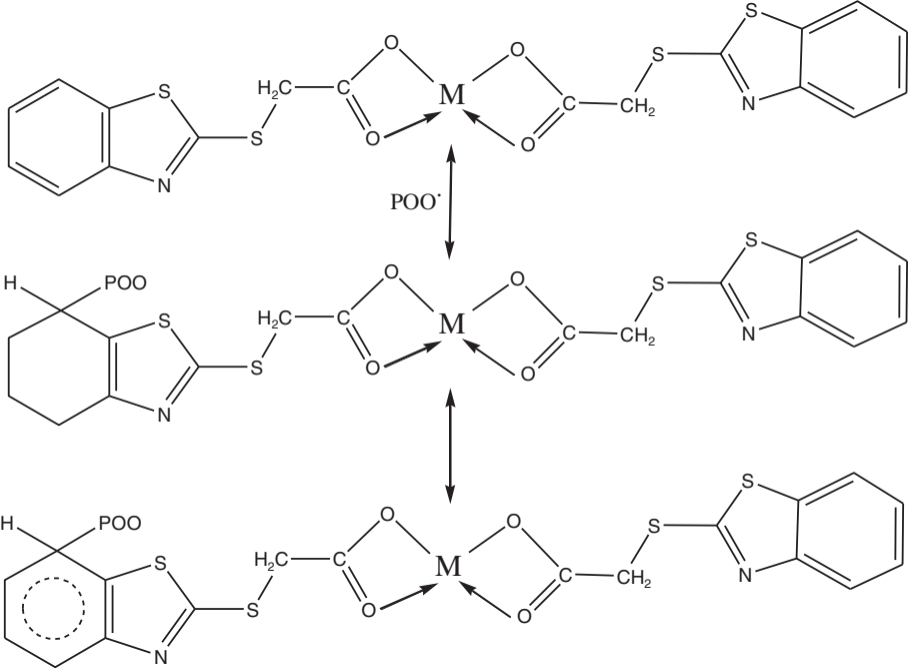

Supplement: Supplementary file 42 — Authors’ original file for figure 42 [file 40064_2013_1415_MOESM42_ESM.pdf]

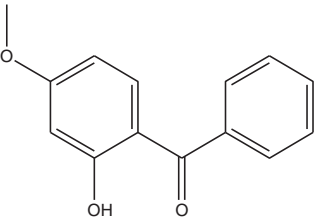

2-hydroxy-4-methoxybenzophenone

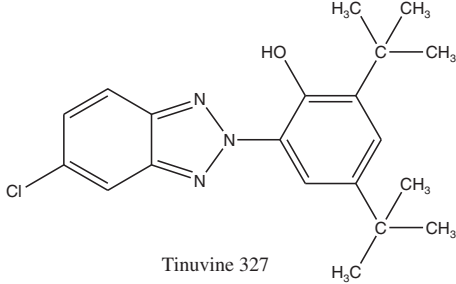

Tinuvin 327

Supplement: Supplementary file 43 — Authors’ original file for figure 43 [file 40064_2013_1415_MOESM43_ESM.pdf]

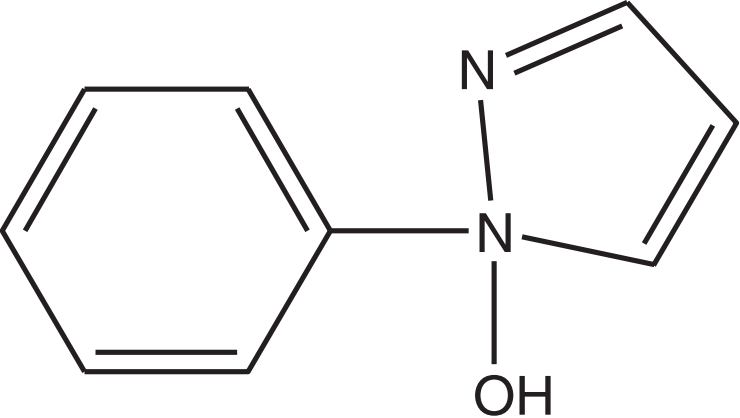

hydroxyl phenyl pyrazole

Supplement: Supplementary file 44 — Authors’ original file for figure 44 [file 40064_2013_1415_MOESM44_ESM.pdf]

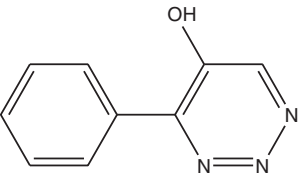

hydroxyphenyltriazine

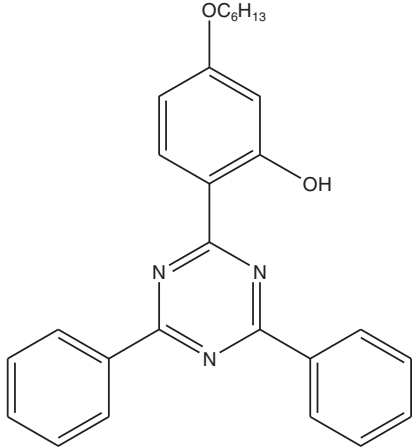

Tinuvin 1577

Supplement: Supplementary file 45 — Authors’ original file for figure 45 [file 40064_2013_1415_MOESM45_ESM.pdf]

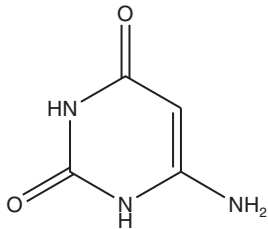

6 aminouracil

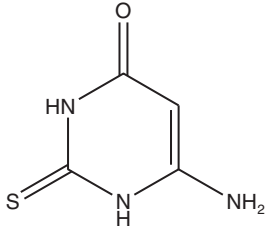

6 aminothiouracil

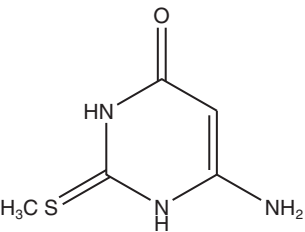

6 amino-s-methyluracil

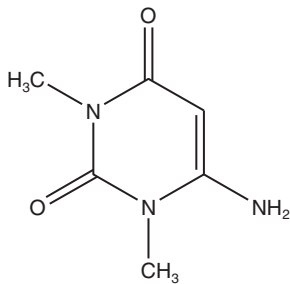

1,3-dimrthyluracil

Supplement: Supplementary file 46 — Authors’ original file for figure 46 [file 40064_2013_1415_MOESM46_ESM.pdf]

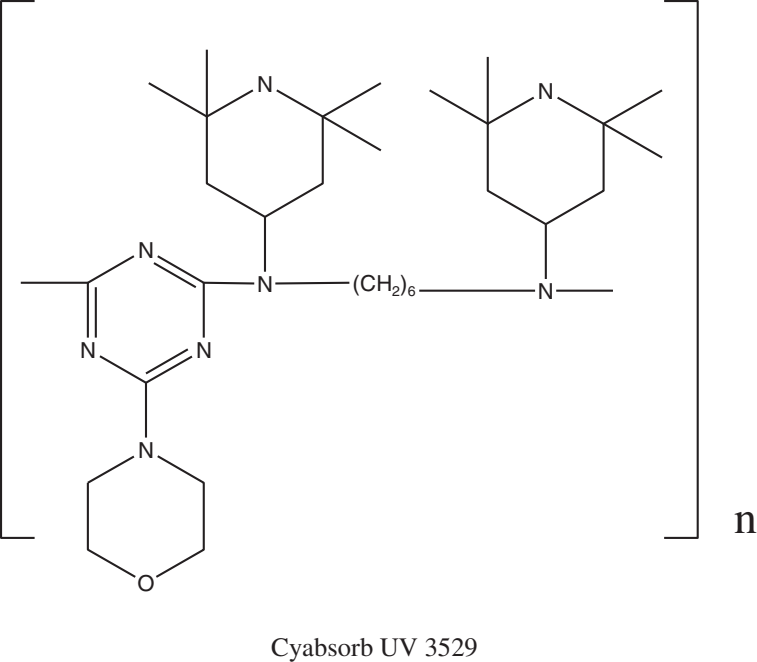

Supplement: Supplementary file 47 — Authors’ original file for figure 47 [file 40064_2013_1415_MOESM47_ESM.pdf]

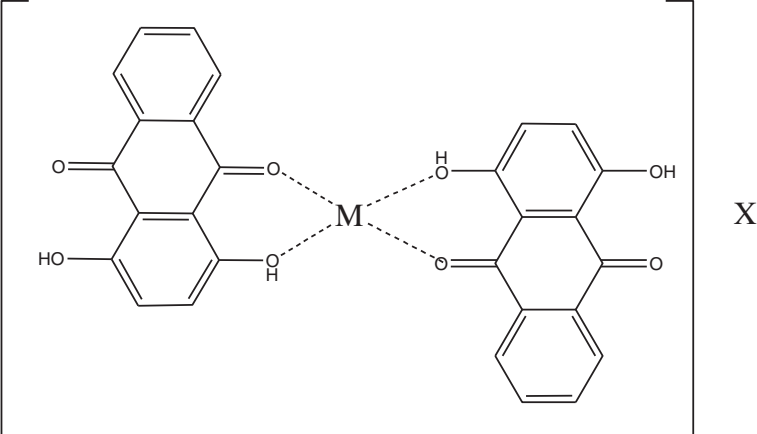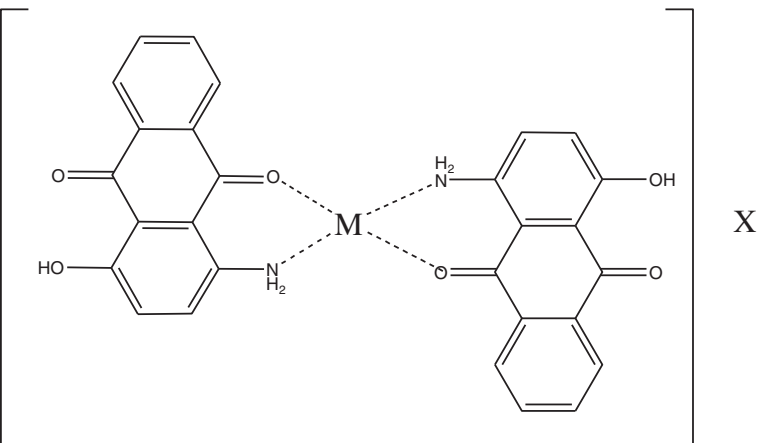

$M=Cu$  ,  $X=Cl$

$M=Vo$  ,  $X=SO_4$

$M=Ni$  ,  $X=(CH_3COO)_2$

Supplement: Supplementary file 48 — Authors’ original file for figure 48 [file 40064_2013_1415_MOESM48_ESM.pdf]

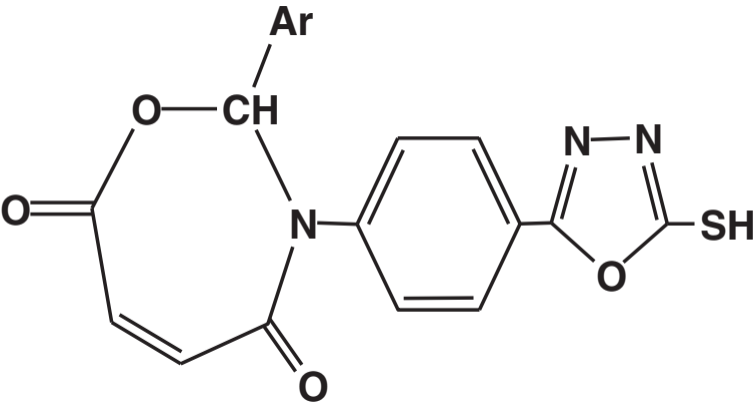

Supplement: Supplementary file 49 — Authors’ original file for figure 49 [file 40064_2013_1415_MOESM49_ESM.pdf]
