# Supplementary material for: Photodegradation and photostabilization of polymers, especially polystyrene: review
Source: Springerplus. 2013 Aug 23;2:398. doi: 10.1186/2193-1801-2-398 (PMC4320144; doi:10.1186/2193-1801-2-398)
Supplement: Supplementary file 50 — Authors’ original file for figure 50 [file 40064_2013_1415_MOESM50_ESM.pdf]

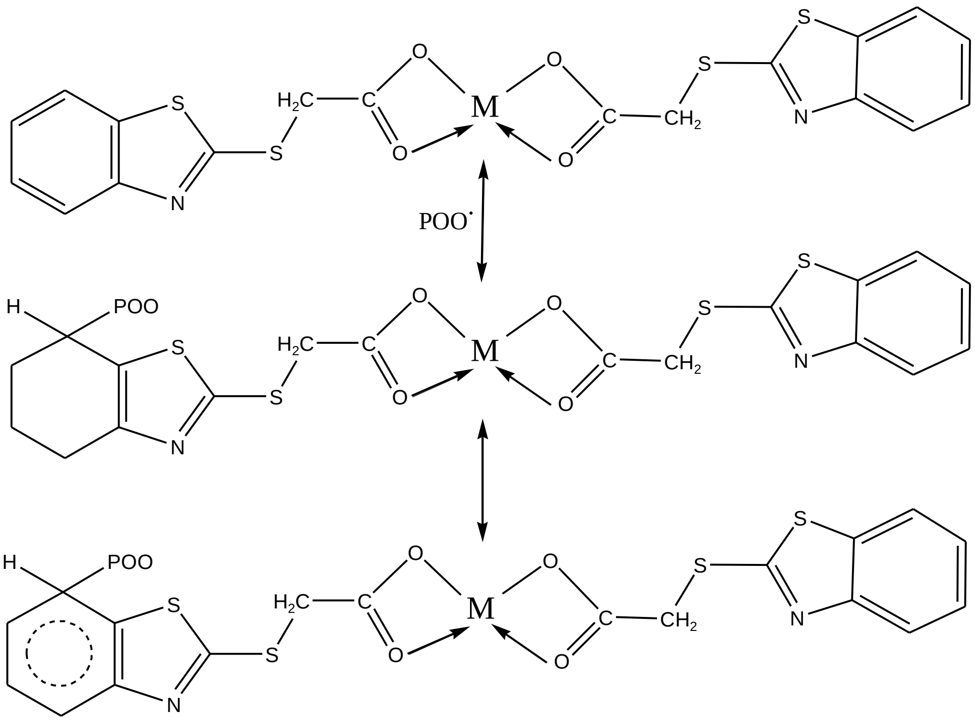

Scheme (7) Suggested mode of photostabilization by carboxylate complexes as radical scavengers.
